# Early Pastoral Economies and Herding Transitions in Eastern Eurasia

**DOI:** 10.1038/s41598-020-57735-y

**Published:** 2020-01-22

**Authors:** William Timothy Treal Taylor, Julia Clark, Jamsranjav Bayarsaikhan, Tumurbaatar Tuvshinjargal, Jessica Thompson Jobe, William Fitzhugh, Richard Kortum, Robert N. Spengler, Svetlana Shnaider, Frederik Valeur Seersholm, Isaac Hart, Nicholas Case, Shevan Wilkin, Jessica Hendy, Ulrike Thuering, Bryan Miller, Alicia R. Ventresca Miller, Andrea Picin, Nils Vanwezer, Franziska Irmer, Samantha Brown, Aida Abdykanova, Daniel R. Shultz, Victoria Pham, Michael Bunce, Katerina Douka, Emily Lena Jones, Nicole Boivin

**Affiliations:** 1 CU Museum of Natural History/Department of Anthropology, CU 218, Boulder, CO 80309 USA; 2 Department of Archaeology, Max Planck Institute for the Science of Human History, 10 Kahlaische Str., Jena, 07745 Germany; 3 Flinders University, Australia, Sturt Road, Bedford Park, South Australia 5042 Australia; 4 National Museum of Mongolia, Juulchiny Str-1, Ulaanbaatar, 21046 Mongolia; 5 Graduate School of Human Development in Landscapes, University of Kiel, Johanna-Mestorf Str 2-6, R.156, D - 24118 Kiel, Germany; 6 Department of Geology and Geological Engineering, Colorado School of Mines, 1500 Illinois St., Golden, CO 80401 USA; 7 Arctic Studies Center, Smithsonian National Museum of Natural History, Washington, D.C., 20560 USA; 8 Department of Philosophy and Humanities, East Tennessee State University, 276 Gilbreath Dr, Johnson City, TN 37614 USA; 9 Institute of Archaeology and Ethnography, Siberian Branch Russian Academy of Science, 17 Lavrentieva Avenue, Novosibirsk, 630090 Russia; 10 Novosibirsk State University, 1, Pirogova Str., Novosibirsk, Russia; 11 Trace and Environmental DNA (TrEnD) Laboratory, Curtin University, Kent Street, Bentley, WA 6102 Australia; 12 Department of Geography, University of Utah, 260 Central campus Drive Room 4625, Salt Lake City, UT 84112 USA; 13 Wyoming Geographic Information Science Center, Department of Geography, University of Wyoming, 1000 E. University Ave., Laramie, WY 82071 USA; 14 Faculty of History, University of Oxford, George Street, OX1 2RL Oxford, UK; 15 Anthropology Program, American University of Central Asia, Aaly Tokombaev st. 7/6, 720060 Bishkek, Kyrgyzstan; 16 Departments of Anthropology and History, McGill University, 855 Sherbrooke Street West, Montreal, Quebec, Canada H3A 2T7; 17 University of Sydney, Australia, Camperdown, NSW 2006 Australia; 18 Department of Anthropology, University of New Mexico, MSC01-1040, Albuquerque, NM 87131 USA; 19 Department of Anthropology, Museum of Anthropological Archaeology, University of Michigan, Ann Arbor, MI 48109 USA

**Keywords:** Archaeology, Archaeology

## Abstract

While classic models for the emergence of pastoral groups in Inner Asia describe mounted, horse-borne herders sweeping across the Eurasian Steppes during the Early or Middle Bronze Age (ca. 3000–1500 BCE), the actual economic basis of many early pastoral societies in the region is poorly characterized. In this paper, we use collagen mass fingerprinting and ancient DNA analysis of some of the first stratified and directly dated archaeofaunal assemblages from Mongolia’s early pastoral cultures to undertake species identifications of this rare and highly fragmented material. Our results provide evidence for livestock-based, herding subsistence in Mongolia during the late 3rd and early 2nd millennia BCE. We observe no evidence for dietary exploitation of horses prior to the late Bronze Age, ca. 1200 BCE – at which point horses come to dominate ritual assemblages, play a key role in pastoral diets, and greatly influence pastoral mobility. In combination with the broader archaeofaunal record of Inner Asia, our analysis supports models for widespread changes in herding ecology linked to the innovation of horseback riding in Central Asia in the final 2nd millennium BCE. Such a framework can explain key broad-scale patterns in the movement of people, ideas, and material culture in Eurasian prehistory.

## Introduction

Horse domestication is widely recognized as a key transformative event in human prehistory. The initial domestication of horses has been linked to major changes in human mobility and social organization, particularly in Inner Asia^1^. Horses have also been invoked to explain continent-scale population movements, such as the spread of some Bronze Age peoples into Europe (e.g.^2^). By the first millennium BCE, the adoption of horses as transport and military animals by settled peoples in China, western Asia, and the Classical World facilitated trade, promoted economic integration, and supported early imperial expansion^3–5^. In early historic North America, where the horse was re-introduced by Spanish colonists after 1492 CE, the newfound availability of long-distance high-speed transport encouraged the emergence of new ethnic groups, including some with economies based on raiding, a transformation which had profound impacts on social and environmental dynamics in the Americas^5^. However, while examples of the ways in which horses transformed human societies abound across the New and Old Worlds, the process by which *Equus caballus* transitioned from a wild animal to the basis of many global pastoral economies is poorly understood. Here, we draw upon the archaeological record of Mongolia and Central Asia and the application of biomolecular methods to newly excavated assemblages from Bronze Age habitation sites to highlight potential causal links between innovations in horse transport and the evolution of pastoral societies in antiquity. 

## Pastoralism in the Eurasian Steppes

The great steppes of Eurasia are characterized not only by broad stretches of dry grasslands, but also a wide range of different environmental and ecological zones – including a diverse mix of deserts, mountain and alpine zones, forest, and productive agricultural valleys. Pastoral herding of domestic animals has been a historically significant lifeway across much of this region, but the species emphasized by particular herding groups within this ecogeographic zone - including sheep, goat, cattle, yak, camel, horse, and others - vary widely according to local environmental conditions^6^ and/or cultural choices^7^. Eurasian domestic livestock species differ in terms of their food and water requirements. Some taxa, like cattle, require high daily water intake and grazing over a limited spatial range, while others, like horse, move frequently over larger ranges to meet their energetic and nutritional needs^8^. These differences in animal ecology underlie differences in the prevalence of specific domestic taxa, the degree of residential mobility practiced, and the role of other subsistence inputs (like grains and agriculture) in regional economies.

In Eurasian steppe areas with limited or inconsistent water availability, horses are particularly useful to pastoralists. Horses thrive on dryland grasses and steppe plants^9^, requiring significantly less water than domestic taxa such as cattle. Horses are able to move substantial distances between water and food sources, as well as dig for their own water during extreme conditions^9^. They are also well-adapted for survival in the harsh steppe winters, possessing strong hooves that enable them to dig through crusted snow and access forage^9^. Ethnographic studies in Kazakhstan note a winter herding cycle that relies on horses hoofing through ice and snow up to a depth of 40 cm, which in turn provides access to buried forage for sheep, goats, and cattle, which are herded in post-horse-grazed fields^10^. In addition, horse meat and milk products are nutritious, providing desirable fatty acids and other benefits for human nourishment^11,12^. Horses are strong and fast. On horseback, herders are able to move quickly and efficiently over long distances even in rough terrain, and are able to tend larger numbers of animals and pasture them at farther distances than would be possible on foot^13^. In some areas of Central Asia, particularly the dry and relatively inhospitable Eastern Steppes of Mongolia, horses today form an essential part of pastoral lifeways^14^.

However, many of the unique abilities which make horses a boon for herders also introduce major logistical difficulties in managing them – difficulties that can only be overcome if herders are capable of fast and efficient movement. Unlike cattle, sheep, and goats, horses range freely and are unlikely to follow the patterns intended by their herders^15^. Moreover, horses may graze up to 16 hours each day to feed their relatively (as compared to ruminants) inefficient monogastric digestive system, and in the wild, may move over a home range of a few to several hundred square kilometers^9^. In contemporary Mongolia, free-range horses largely organize themselves in line with their natural social structure, with a lead stallion and a harem of mares, geldings, and juveniles^16^. Most horses are gathered by herders only for particular purposes (for example, castration, milking, and breeding) and at particular times. Managing large numbers of domestic horses thus requires the ability to keep track of animals that may range over great distances, a task that is in most cases nearly impossible on foot. During the 20^th^ century, families and groups that specialized in domestic horse herding practiced the widest-ranging seasonal migrations, sometimes moving in excess of 200 km a year^17^. Due to the logistical challenges in horse-based pastoralism, some scholars have argued that managing large numbers of horses as livestock was impossible without the ability to ride them (e.g.^13^).

## Understanding Early Horse Domestication and Transport

**Figure 1:**
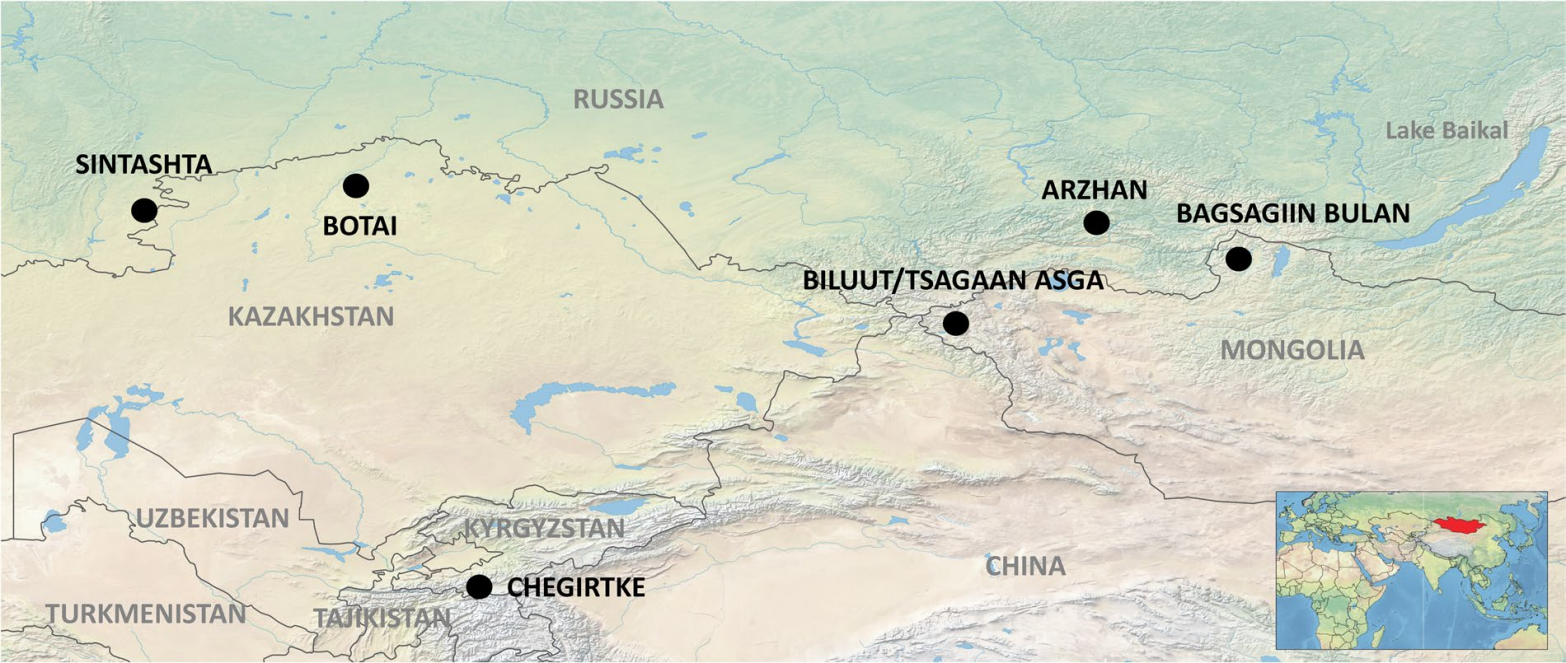
Selected archaeological sites discussed in the text.

Characterizing the origins of horse domestication thus requires careful consideration of this species’ potentially intertwined role as both livestock and transport. The oldest evidence for horse domestication can be traced back to the Botai culture (Fig. 1), found in the Trans-Ural region of northern Kazakhstan and southern Russia and dated to ca. 3500 BCE. Faunal assemblages linked to this culture are dominated by horse bones; other relevant discoveries include ritual burial pits, possible corral structures, a horse-bone tool industry, and evidence for concentrations of manure^18,19^. Lipid residues on ceramics suggest that Botai people may have used horse milk, and damage to some horse lower premolars suggests that Botai horses may have been harnessed or “bitted” with a mouthpiece^19^. On the basis of the Botai evidence for horse management, Anthony^1^ argued that horseback riding had emerged by ca 3000 BCE or before. This chronology, with the assumption of horse riding as a driving cultural force, has subsequently underpinned many high-profile models for Eurasian social transformations during the late Neolithic and Early Bronze Age, most notably the apparent migration of steppe people into eastern Europe and other large-scale population movements during the late Holocene^2,20^.

However, advances in biomolecular archaeology have overturned crucial elements of the story of horse domestication at Botai and in so doing have undercut the hypothesis of broad-scale, horse-based population movements during the early Bronze Age. Ancient DNA indicates that horses at Botai were in fact *Equus przewalskii* – the domestic horse’s closest extant relative – and not the progenitor of *Equus caballus*^21^. Recent large-scale studies in human genomics have also revealed no genetic connections between Botai people and cultures involved in early steppe migration events (Yamnaya, Afanasievo)^20^, and shown that the geographic impact of these events was limited^20^. These findings raise questions about the early prehistory of domestic horse use and necessitate a reconsideration of the economic role of horses in ancient Central Asia.

The earliest incontrovertible evidence for the use of horses as transport dates to ca. 2000 BCE, to chariot burials of the Sintashta culture (Fig. 1). These burials typically comprise paired ritual horse head-and-hoof burials along with bridle equipment and sometimes physical chariot remains or wheel impressions (e.g.^22,23^). Depictions of horses from the Near and Middle East suggest horses were only very rarely ridden prior to the end of the second millennium BCE, and when they were, control was unreliable and dangerous^24^, perhaps undertaken primarily for sport or athletic demonstration^25^. The brutal, spiked design of early chariot cheekpieces, and heavy evidence of use wear on horse teeth suggest control must have been a serious challenge for the first charioteers^26^. While many scholars – especially those with experience on horseback – are intuitively attracted to the idea that riding preceded cart or chariot traction, Dietz^27^ argues compellingly on the basis of temperament, musculature, and behavior, that controlling early domestic horses as a chariot team would have been markedly easier than mounted riding. Although the question is far from resolved, many influential scholars (e.g.^25,28^) support the idea of this ‘cart before the horse’ model, placing the development of mounted riding and associated social transformations in Central Asia towards the second half of the second millennium BCE. Perhaps owing to the greater efficacy chariots provided in control over early domestic horses, the two spread together in tight association across much of the Old World, reaching Egypt by the middle second millennium BCE^29^ and China by the late second millennium BCE^30^.

By the early first millennium BCE, archaeological and historical records attest to the emergence of both mounted horseback riding and specialized horse-based ways of life in Inner Asia. Historical records refer to horse-mounted warriors in western Asia by the 8th century BCE, while archaeological finds from localities like Arzhan 2 in southern Tuva show specialized horse equipment (bronze snaffle bits) and equine vertebral pathologies linked with mounted riding in Central Asia by the late 9th century BCE^31^. Drews^25^ and others link this period with the initial adoption and spread of mounted horseback riding in Central Asia.

## Horses and Bronze Age Economic Transitions in Mongolia

Recent archaeological work has traced the origins of Mongolia’s horse-based pastoral economy as far back as the late Bronze Age (ca. 1500–700 BCE), to archaeological sites of the Deer Stone-Khirigsuur (DSK) complex^32,33^. Beginning around 1200 BCE, monuments and burials often surrounded by dozens, hundreds, or even thousands of associated horse sacrifices radiated from the Khentii Mountains in the east to as far west as Kazakhstan and Issyk-Kul in Kyrgyzstan, and from Tuva and southern Russia as far as northwestern China^33^. Osteological features on DSK horse burials indicate that these animals were bridled and heavily exerted^34,35^ and were probably used for mounted riding^36^. People in the DSK culture group appear to have experimented with veterinary care and dentistry^37^ and managed horses in breeding herds^38^. Although only a few DSK habitation sites have been identified, this period is associated with the first archaeofaunal evidence for dietary exploitation of horses^39^. In addition, human skeletal remains from this period indicate a major increase in entheseal changes to male human skeletons in regions of the body linked with horseback riding, including the hips and elbows, particularly those of the left-hand side^40^, which is often favored by Central Asian horse riders today, and appears to have been similarly favored in antiquity^36^. These developments appear to have been concurrent with the initial geographic spread of horses to the Central Plains of China. Without comparative archaeological data from the preceding centuries, however, it is difficult to assess whether the fluorescence of horse culture in Mongolia was linked with an actual economic change in the use of horses^33^.

The identification of such comparative data, however, has proven remarkably challenging due to the lack of domestic archaeological assemblages^41^. The earliest circumstantial evidence for herding lifeways in Mongolia can be traced to ca. 3000 BCE, when burials attributed to the Afanasievo cultural horizon can be found in some areas of western and central Mongolia^42,43^. These tombs contain the remains of disassembled carts as well as sheep and cattle bones, findings that has been drawn upon to infer that western animal domesticates were likely introduced to the Eastern Steppes of Mongolia at this time^44^, although some scholars suggest that domestic sheep may have already been present in some areas of northern China as early as ca. 3700 BCE^45^. Petroglyphs depicting tethered cattle, cattle carts, and horses have been found depicted on stones used to construct ritual and funerary sites from the Middle Bronze Age Chemurchek culture in western Mongolia^46^ and at least one of these features, dated to the early second millennium BCE, contains equine skeletal remains^46,47^.

Here we present findings from our own archaeological survey and excavation in Mongolia as well as reanalysis of previously excavated material. We excavated two domestic habitation sites dating to the early Bronze Age – the first such sites pre-dating the second millennium BC in Mongolia – including one structure with stratified and securely dated faunal remains. We also analyse faunal material from a third Bronze Age site, previously reported by Fitzugh and Kortum^48^, but not previously analysed. We undertake collagen mass fingerprinting and ancient DNA analysis of recovered faunal assemblages, along with reanalysis of previously excavated materials, to assess chronological patterns in domestic horse use and pastoral economies in Mongolia. We draw on these findings to examine issues of horse transport and explore the broader implications of our data for understanding cultural dynamics across ancient Eurasia.

## Materials and Methods

We conducted archaeological research at three localities: (1) the newly-identified, unstratified habitation of Bagsagiin Bulan, near Soyo Tolgoi in the Darkhad basin of northern Mongolia; (2) a second newly discovered and stratified habitation site at Tsagaan Asga, in western Mongolia’s Altai Tavan Bogd National Park adjacent to Dayan Lake, and (3) the previously excavated, nearby habitation site of Biluut on Khoton Lake, also in Altai Tavan Bogd National Park (Fig. 1). Following this, we conducted meta-analysis of published archaeofaunal assemblages from across the central Asian Bronze Age to assess broad chronological trends in the occurrence and frequency of domestic horse remains.

We conducted excavations at Tsagaan Asga and Bagsagiin Bulan using a modified Harris Matrix system, excavating units according to cultural and stratigraphic layers. Using a Leica Total Station, we documented the precise location of each animal bone and artifact as well as each piece of charcoal greater than 2 mm in maximum diameter, and excluded those specimens that lacked clear stratigraphic information (such as those located in disturbed areas) from our laboratory analysis. Stratigraphic profiles for excavations at both localities, as well as detailed analysis of geologic context, are provided in the Supplementary Appendix (S2 and S4). Using a Nikon D610 FX camera in conjunction with AgiSoft Photoscan Pro, we produced a high-resolution 3D photogrammetric model of each site prior to excavation and at the terminus of each stratigraphic layer. We sifted all sediment using a 1/16” (1.5875 mm) mesh, and from each context also selected 40 L of sediment for flotation using 0.355 mm geologic sieves. Radiocarbon samples were selected from animal bone and charcoal remains from within cultural features (the wall at Tsagaan Asga, and the central hearth at Bagsagiin Bulan), and processed at the Center for Isotope Research at the University of Groningen, Netherlands. Radiocarbon dates were calibrated using the INTCAL13 calibration curve via OxCal.

For each specimen, we examined each specimen for surface modification and other indicators that would provide insight into taphonomic history, taphonomic analysis and, where possible, performed species identifications using comparative faunal collections at the Max Planck Institute for the Science of Human History in Jena, Germany. To assign species using the collagen mass fingerprinting technique Zooarchaeology by Mass Spectrometry (ZooMS), we demineralized and extracted 10 mg of bone using 50 mM ammonium bicarbonate (Sigma-Aldrich) pH 8, following the protocol outlined by van Doorn *et al*.^49^. The collagen was digested into component peptides using trypsin (Pierce), and purified using Pierce C18 tips (Thermo Scientific), eluting in 50% acetonitrile (Sigma-Aldrich) and 0.1%TFA (Sigma-Aldrich). For the small assemblage from Bagsagiin Bulan that failed to produce recognizable collagen spectra using this protocol, we repeated our analyses using a more stringent extraction protocol^50^. For these seven specimens, we demineralized and extracted 10 mg of bone using 1 ml of 0.5 M hydrochloric acid (HCl). The bone powder was refrigerated in this solution overnight (18 hours), before being removed into a 30kDA ultrafilter and centrifuged at 3700 rpm for one hour. After this process was complete, we added 500 µL of ammonium bicarbonate solution, and then centrifuged for one hour. After mixing extracted collagen with ammonium bicarbonate, this solution was digested into component peptides using trypsin (Pierce) and diluted using 50% acetonitrile (Sigma-Aldrich). Samples were then spotted on Bruker AnchorChip or Ground Steel plates with Bruker Peptide Calibration Standard in calibration spots directly neighbouring the samples. Mass spectrometric analysis of mass/charge ratios was conducted using a Bruker Autoflex Speed LRF MALDI-TOF in the ZooMS Laboratory at the Max Planck Institute for the Science of Human History in Jena, Germany. The acquisition used the following parameters: 4000 laser shots at 50–60% intensity (50 shots per spot), mass range 600–3500 Da, reflector mode. Identifications were made using published reference spectra from a Eurasian mammals database^51^ using FlexAnalysis software, and are reported according to the level of taxonomic specificity. In some cases (e.g., sheep vs. muskox and chamois), species that were not necessarily separable on the basis of observed peptide markers were inferred on the basis of known habitat distribution. All peptide marker data are provided in Table S1 and additional details regarding MALDI-TOF parameters are provided in section S2.

For ancient DNA analysis, one sample of 25 bones (aDNA sample 1) and four samples of 50 bones each (aDNA samples 2–5) from Peat Valley 1 (Biluut 3–3) were analysed using bulk bone metabarcoding^52^. First, each sample was ground on a Retsch PM200 Planetary Ball Mill at 400 rpm and incubated overnight in digestion buffer (0.25 mg Proteinase K + 1 mL 0.5 m EDTA) at 55 °C. Subsequently, the supernatant was concentrated to 50 µL in a MWCO 30,000 Vivaspin 500 column (Sigma-Aldrich) and purified in a MinElute PCR Purification column as in Seersholm *et al*.^53^. Lastly, two mitochondrial assays targeting mammals (Mam16S^54^) and all vertebrates (12SV5^55^) were amplified and sequenced on the Illumina MiSeq platform in the Trace and Environmental DNA (TrEnD) Laboratory at Curtin University, Perth, Australia. After sequencing, filtered and denoised DNA reads were queried against the NCBI nt database^56^ using megablast^57^ and, subsequently, each read was assigned to the taxonomic node of the best blast hit(s) using the script blast_getLCA.py (https://github.com/frederikseersholm/blast_getLCA). Complete results are presented in Supplementary Appendix (Section S7).

For our meta-analyses, we aggregated published faunal data to calculate percent number of identified species (%NISP) for Central Asian archaeological sites. We used NISP rather than minimum number of individuals (MNI) to avoid the severe aggregation problems associated with MNI (e.g.^58^), which can be particularly problematic in meta-analyses such as this one^59^. When raw counts were available, we used all specimens identified to the genus *Equus* divided by the total number of identified specimens to generate percentages; in other cases only %NISP was reported in original publications and this value was reprinted here. For a single case (from^60^), %NISP was back-calculated from published graphs using the freeware measurement program ImageJ. It should be noted that as these original publications varied widely in their methods and reported detail, and because these taxa may be challenging to distinguish morphologically, the summarized data may include non-domestic equids such as khulan (*Equus hemionus*) or Przewalski’s horse (*E. przewalskii*). Chegirtke are based upon previously published taxonomic estimates made using ZooMS^61^, while other percentages were done through comparative archaeozoological study by the original investigators. Frequencies for newly analyzed assemblages (Tsagaan Asga, Bagsagiin Bulan, and Biluut) are reported using both comparative archaeozoology and ZooMS. This combination of methods means that any specific instances of horse in the record that we identify should be interpreted with caution; we use these data not make claims about the presence or absence of riding horses in any particular assemblage, but only to instead identify broad-scale changes over time^62,63^, with an eye to assessing the dietary role of horses in pastoral economies.

Site locations were approximated in qGIS after georeferencing published maps in Eregzen^43^ and placed on a digital elevation model and hillshade acquired from Rutgers University Department of Marine and Coastal Sciences.

## Results

### Bagsagiin bulan

**Figure 2:**
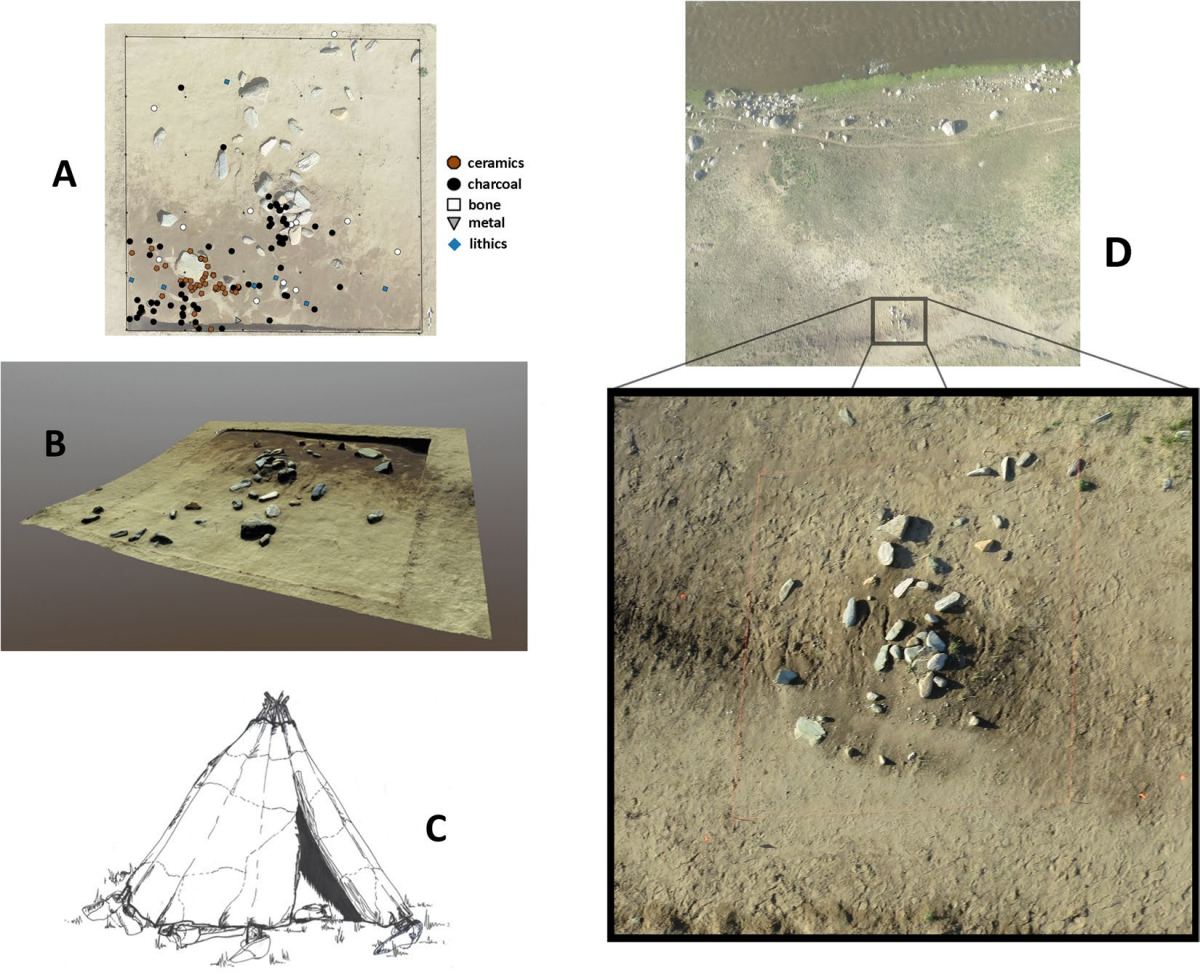
(**A**) Habitation structure and occupation surface at Bagsagiin Bulan, in northern Mongolia. (**B**) Structure shown as 3D photogrammetric model. Central hearth feature is eroding from the bank, while the upper third of the structure remains along with upright support stones. (**C**) Artist’s reconstruction of habitation structure at Bagsagiin Bulan (Drawing by V. Pham). (**D**) Aerial photograph of riverbank and structure, with excavation area highlighted. View to the north, facing the Hugiin Gol. Image by W.Taylor and N. Case.

During archaeological survey at Bagsagiin Bulan (51°00'54.4″N 99°13'02.9″E) during the summer of 2016, we identified a circular structure consisting of a ring of upright stones, probably acting as pole supports, surrounding a central hearth feature (Fig. 2A,B). This structure was most likely a kind of pole-tent (Fig. 2C, Supplementary Appendix S2). Based on the spacing and size of the uneroded structure, the original feature was approximately 4 m in diameter with between 15–20 upright poles. A single, flat heavy stone was laid against one of the upright stones, as is typical in contemporary *orts* (teepee-style habitations in the Mongolian taiga), to weigh down wall coverings (Appendix S2). A post hole indicates that some kind of exterior support structure was likely present. The site was built on a river terrace that, based on a geomorphological study of the region, likely formed ca. 2500 BCE (~4.5 ka, Supplementary Appendix S2). The structure was set into undisturbed sand, and its remaining components were gradually buried by a combination of aeolian and cultural deposition. Localized areas of the site were also disturbed by rodent activity and large frost polygons, the latter associated with permafrost or seasonal frost (Appendix S2).

By the time of its excavation in 2017, most of the feature had been exposed and eroded by the modern riverbank. Our team recovered Bronze Age ceramics, microblades and microblade cores (Appendix S2C), as well as charcoal from within and around the feature at a depth of ~20 cm below the modern surface. A radiocarbon date on charcoal from the central hearth feature places the burning activity at ca. 2500 BCE (3285 +/− 15 14 C YBP, ca. 2626–2495 cal. BCE, Appendix S5), corresponding broadly to the estimated age of the river terrace formation (Appendix S2).

**Figure 3:**
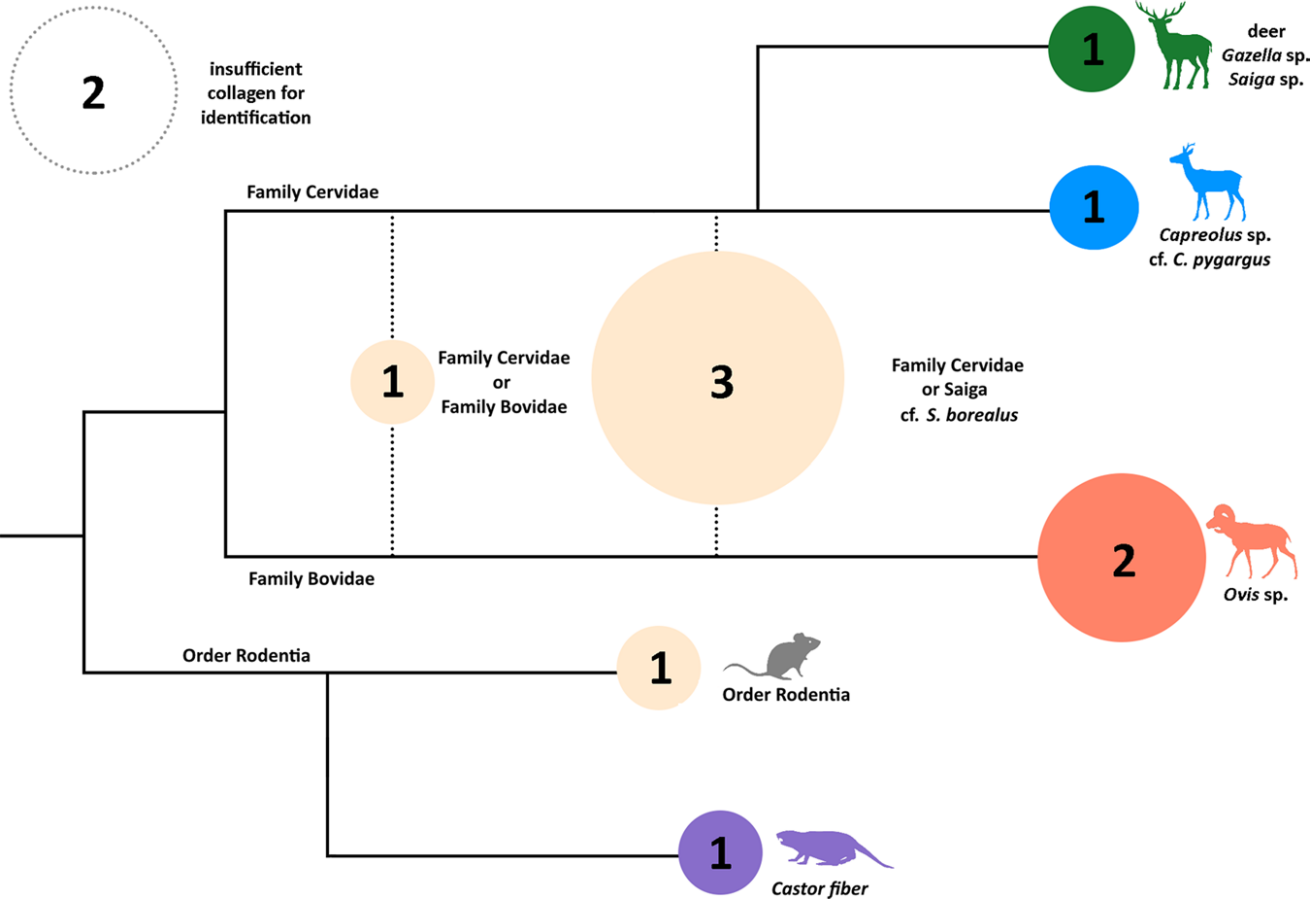
Zooarchaeology by Mass Spectrometry (ZooMS) taxonomic identifications overlain on a schematic of a cladogram for bone remains from Bagsagiin Bulan. Specimens with conclusive markers are shown in color (blue, red, green, purple), while those with missing markers are shown in tan according to the greatest possible level of taxonomic specificity. Circles have been scaled to size according to quantity of identified specimens in each category and inset numbers refer to the number of specimens identified. Dotted circle in top left shows specimens with insufficient collagen for analysis.

A small number of bones were recovered from within the structure’s interior at a depth of >20 cmbd. While these bone fragments are very small and unidentifiable by traditional archaeozoological methods, we employed the peptide fingerprinting technique, which uses taxon-specific masses of collagen peptides as a method of species identification^60^. Using ZooMS, we were able to identify roe deer (*Capreolus* sp. n = 1 fragment), deer or saiga/gazelle (n = 1 fragment), beaver (*Castor* sp. n = 1 fragment), and *Ovis* (n = 2 fragments, Fig. 3) in the assemblage. As only one species of roe deer occurs naturally in the region, this specimen is almost certainly the Siberian roe deer, *C. pygargus*.The *Ovis* specimens may represent either wild Argali sheep (*O. ammon*), or the domesticated *O. aries*. However, all three of the individual animal bones selected for ^14^C dating (roe deer, deer/gazelle/saiga, and indeterminate cervid) yielded a late historic or modern date, while charcoal from the top of the cultural layer (outside the structure) dated to the Iron Age. This suggests that the original cultural deposit was exposed at the surface in prehistory, and/or influenced by the downward transgression of later, historic faunal material. As a result, no conclusions can be drawn about Bronze Age subsistence from the identified bone assemblage. Nonetheless, we did discover carbonized *Chenopodium* seeds from flotation samples recovered from sediments containing a concentration of charcoal adjacent to the structure, and from within the hearth feature (Appendix S6). We suspect that the frequency of these endozoochoric seeds in this feature could reflect the practice of dung burning, as argued for in other similar contexts (see Appendix S6), although it is unclear to what degree this material may relate to the original use of the structure.

### Biluut

**Figure 4:**
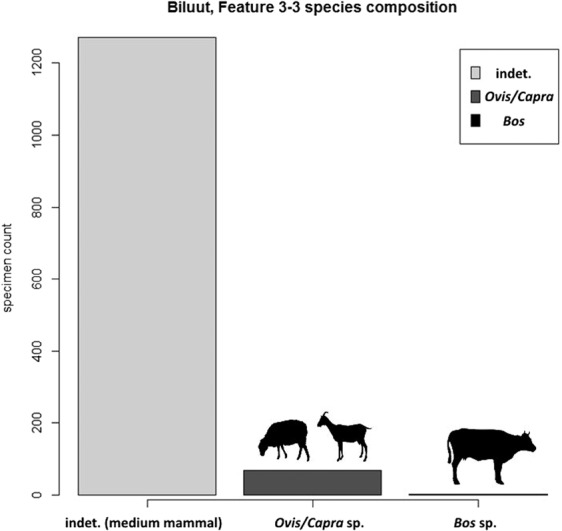
Species composition of calcined bone fragments from Biluut, Peat Valley 1 (non-faunal data originally reported in Fitzhugh and Kortum 2012), based on new morphological identifications by T. Tuvshinjargal. The assemblage was unanalyzed in the initial report, and ZooMS/aDNA efforts yielded no collagen or DNA for biomolecular identifications.

We also reanalyzed one of the few previously identified Mongolian faunal assemblages dated to the early/middle Bronze Age. Analysis of this assemblage also suggests the presence of pastoral domesticates in western Mongolia during the early Bronze Age, and shows no evidence for dietary exploitation of horses. Biluut (referred to as either Peat Valley 1, or Biluut 3.3, 48°39.165′, E88°21.588′) consists of a double-walled rectilinear stone structure with a central hearth and four linear stone-lined trough features associated with microblades and lithic debitage. Although the site was covered by only a shallow turf layer and had poor faunal preservation, the central hearth pit yielded an abundance of fragmented and highly calcined mammalian bones, which were originally hypothesized to be marmot or rabbit^48^. This feature was radiocarbon dated to ca. 2136–1907 BCE on the basis of charcoal from within the central hearth (Beta-306035, 2 sigma calibrated range^48^), although it should be noted that the broader site area has many apparent components, and has not been exhaustively excavated. Using morphological comparisons, we identified that nearly all of the recognizable specimens (n = 61) were in fact sheep or goat (*Ovis* sp./*Capra* sp., Fig. 4). Two additional specimens were identified as belonging to the genus *Bos* (cattle, yak, or other bovid). ZooMS analysis and bulk bone DNA metabarcoding of 25 bones from this structure^52^ that appeared to be the least calcined did not yield identifiable collagen or endogenous DNA - likely due to the site’s shallow burial and the severity of apparent burning.

### Tsagaan Asga

**Figure 5:**
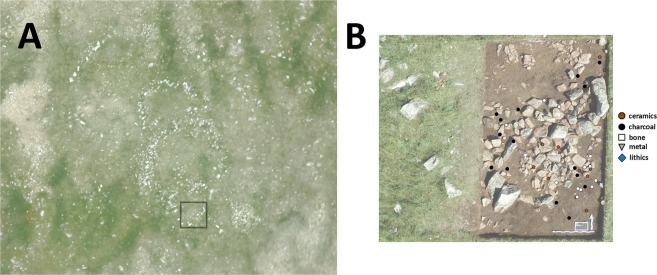
(**A**) Aerial view of structure at Tsagaan Asga with excavated area highlighted, and (**B**) planview of excavated structure showing position of artifacts. Image by W.Taylor and N. Case.

**Figure 6:**
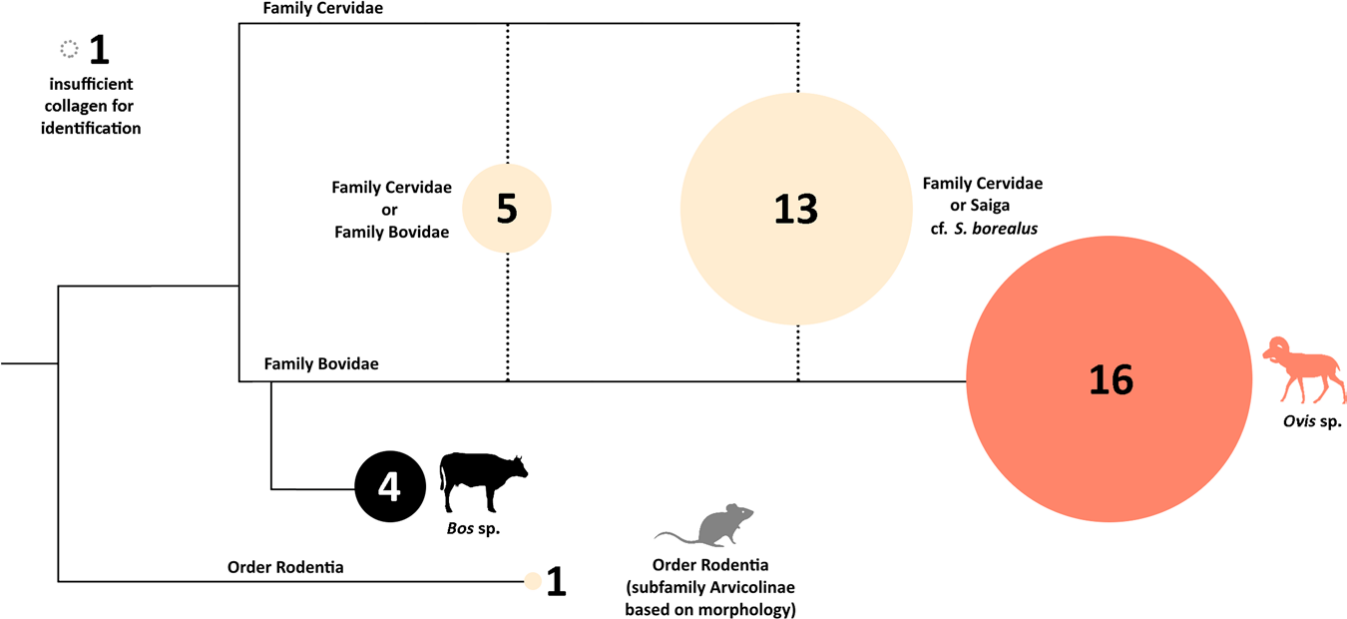
Zooarchaeology by Mass Spectrometry (ZooMS) taxonomic identifications overlain on a schematic of a cladogram for bone remains from Tsagaan Asga. Specimens with conclusive markers are shown in color (blue, red, and black), while those with missing markers are shown in tan according to the greatest possible level of taxonomic specificity. Circles have been scaled to size according to quantity of identified specimens in each category. The unknown rodent was identified based on morphology to the subfamily of Arvicolinae (voles, lemmings, and muskrats). Dotted circle in top left shows specimens with insufficient collagen for analysis.

Finally, during new archaeological surveys at the nearby locality of Tsagaan Asga (48°30′00.8″N 89°00′50.7″E), located on the eastern edge of Altai Tavan Bogd National Park near the margin of Dayan Nuur, we discovered a multi-room stone structure near a modern herding camp on an ancient lake terrace (Appendix S4). Unlike Biluut, which was largely exposed to the surface, the structure at Tsagaan Asga was overlain by 10–20 cm of protective sediment. Radiocarbon dates on sheep bones recovered from within buried cultural deposits inside the structure (Fig. 5) place its occupation concurrent with ritual features known in the area at ca. 1600 BCE (3308 +/− 15 14 C BP, ca. 1626–1530 cal. BCE 2-sigma range). Our test pits revealed bronze slag, Bronze Age ceramics, and a small assemblage of fragmented animal remains, which were largely unidentifiable by standard morphological analysis. Using ZooMS^64^, we determined that most of the large/medium mammal taxa that could be identified to genus level (n = 16) were *Ovis* (Fig. 6), and four were *Bos* sp. A further 13 were missing a necessary peptide marker to distinguish them from wild cervids like deer, but are also likely *Ovis*, based on the absence of other identifiable species in the assemblage with similar markers. The absence of other wild game and the emphasis on a single taxon, here as well as at Biluut, suggests these are domestic animals. This reasoning, combined with the similarity of the Tsagaan Asga structure to habitation features in northwest China^65^, suggests both these sites are, in fact, early pastoral occupations.

### Meta-analysis of inner asian faunal assemblages

**Figure 7:**
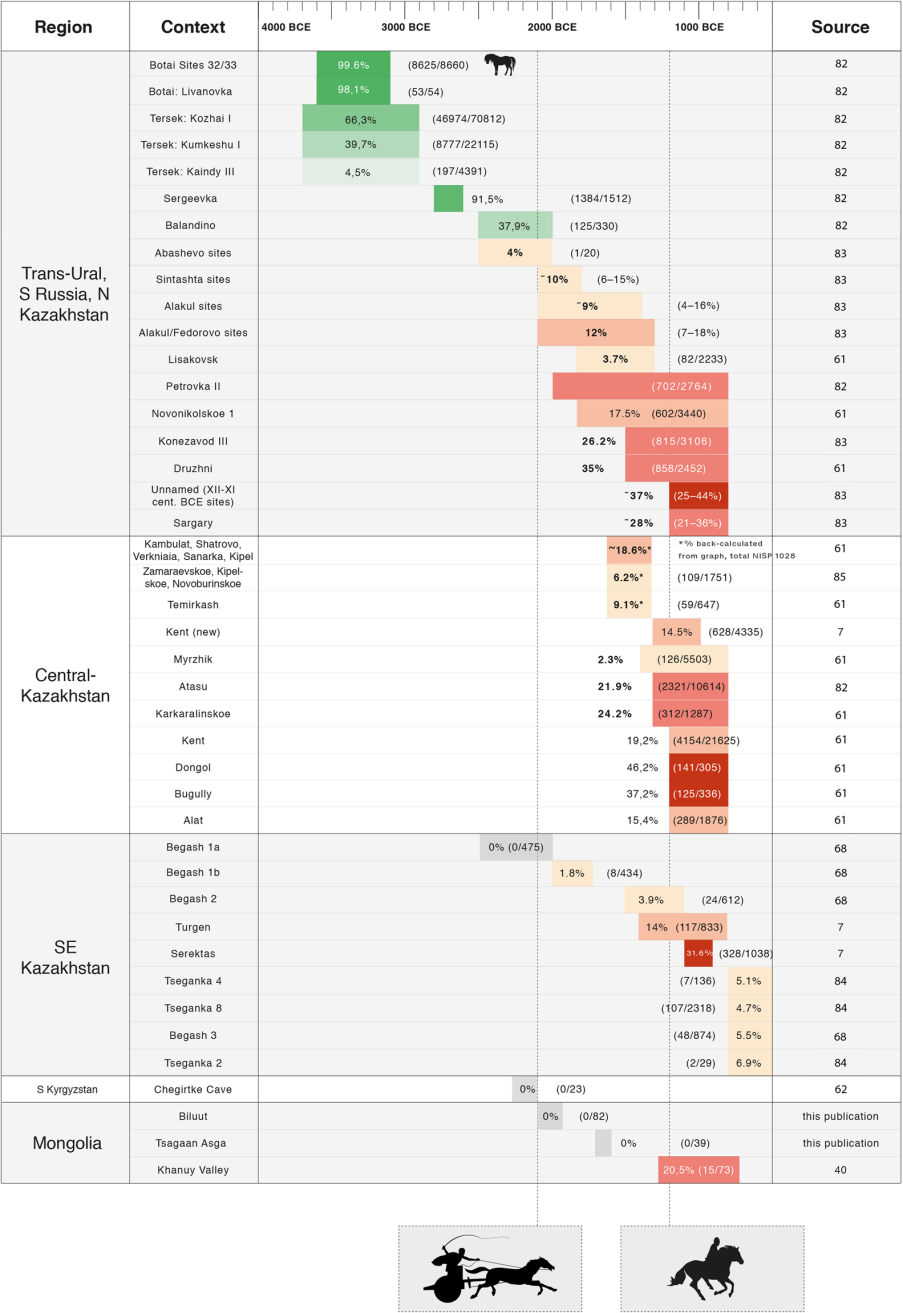
Frequency (%NISP) of horse remains at Bronze Age and Early Iron Age Central Asian archaeological assemblages, as compared to the earliest direct evidence for chariot use at Sintashta (ca. 2000 BCE) and horse riding in the DSK culture (ca. 1200 BCE). Green color indicates those with equivocal domestication status^82–85^.

The broader archaeofaunal record of Eurasia suggests important horse-related transformations to pastoral economies during and the end of the second millennia BCE (Fig. 7). At Eneolithic and Bronze Age sites from Russia, the Black Sea region, and northern Kazakhstan, equid bones of uncertain species and domestication status sometimes occur in high frequencies. For example, at Yamnaya-culture sites from the Ukraine, dated to the end of the 4^th^ millennium BCE, equid bones comprise a meaningful percentage of dietary assemblages^66^. However, these animals may be *E. przewalskii*, as at Botai, or other hunted equids, and some scholars have expressed doubt as to their domestic status. Pre-Sintashta sites in NE Kazakhstan and Russia from the 3^rd^ millennium BCE such as Sholpan and Grigorievka also show non-horse domestic animal taxa and meaningful frequencies of equid remains^20^. Future work will need to assess whether equids from these localities are early *E. caballus, E. przewalskii*, or another wild lineage of horse, and assess their relevance for horse domestication (Fig. 7).

In any case, horses comprise a negligible %NISP in most Central Asian pastoral archaeozoological assemblages before the second millennium BCE (Fig. 7). In fact, in pastoral archaeological assemblages from southerly regions of Central Asia (Kyrgyzstan and southern Kazakhstan) predating the Sintashta chariots, horses are entirely absent^60,67^. The faunal assemblage from the early Bronze Age of Chegirtke Cave (ca. 2300–2100 BCE) in the Alay Valley of southern Kyrgyzstan (Fig. 1), for example, contains a predominance of sheep and some goat and cattle, but no domestic horse remains^61^. After 2000 BCE, horses appear more widely in Central Asian pastoral assemblages but remain low in frequency throughout the second millennium BCE, typically comprising less than 10% of the total identified specimens (Fig. 7). Although, chronological control is sometimes poor in these published assemblages (e.g. Petrovka II, 17th-9th centuries BCE), no published archaeological assemblages from the mid-second millennium BCE show frequencies of more than 20% NISP horse remains (Fig. 7). In assemblages dating to the end of the second millennium BCE and afterwards, the upper range of observed frequency of horse remains at archaeological sites changes dramatically. At these later sites, while horse bones are still found in low frequency at some sites, at others they often comprise between 25–50% of the faunal specimens (Fig. 7). Directional biases^63^ are unlikely to be causing such a dramatic increase in representation.

## Discussion

The extreme rarity of stratified and directly-dated faunal assemblages linked to the early pastoral period make our new discoveries especially significant in understanding the transition to herding economies. Across Mongolia, consistent and severe wind deflation and aridity conspire to produce a near-total absence of buried habitation sites^68^, while those which are recovered typically lack associated or dated faunal remains. Consequently, while our excavated structure at Bagsagiin Bulan does not have securely associated faunal material, it nonetheless provides a rare, direct window into the lifeways of people at the transition to herding economies in eastern Inner Asia. More importantly, although our newly identified assemblages at Tsagaan Asga and Biluut are small and conclusions must be drawn cautiously, these data points provide the very first securely dated insights into the economic use of domestic animals prior to the final Bronze Age.

Archaeological materials from Bagsagiin Bulan show the presence of stone tools and a tent-like structure, hinting at a mobile lifeway rooted in earlier hunter-gatherer traditions ca. 2500 BCE. Herding is practiced in the Darkhad Basin area around Bagsagiin Bulan today, but the region straddles the steppe-boreal forest ecotone, and might be most appropriately characterized as part of broader Baikalia - a well-watered area with rich wild game, timber, and natural resources. Although our excavations provided no faunal data reliably associated with the site’s occupation, across the Russian border to the north, recent work demonstrates that hunting and gathering persisted along the shores of Baikal until a cultural disruption associated with the incursion of pastoralism during the late second and early first millennium BCE^69,70^.

Our newly excavated and analysed archaeofaunal assemblages from western Mongolia provide direct evidence for pastoral economies during the Middle Bronze Age. Faunal assemblages from Biluut and Tsagaan Asga yielded only *Ovis*, indeterminate caprine, or *Bos* remains. The strict focus on these taxa suggests that pastoral herding was practiced in the Mongolian Altai by the late third and early second millennium BCE. Although these two localities are the only domestic sites dated to the Bronze Age in the Mongolian Altai yet known, horse bones have been recovered from within Chemurchek ritual sites such as Poligon I, dated to ca. 1720 BCE (3370 +/− 350 cal BCE, median date 1718 cal BCE, ca. 2626–833 cal BCE 2-sigma calibrated range^46^). DNA analysis will be necessary to assess whether these remains came from the lineage of the domestic horse (*E. caballus*) or other lineages of wild or proto-domesticate animals. However, their occurrence in this ritual context provides compelling reason to suspect that pastoral people had domestic horses at the time that these sites were occupied, during the early second millennium BCE. Nonetheless, faunal assemblages from Tsagaan Asga and Biluut provide no support for the idea that horses made a meaningful dietary contribution to the diet of Early and Middle Bronze Age pastoralists.

The data from these three sites point to incipient pastoral cultures building sturdy occupational structures, and using horses in occasional ritual contexts – but not yet any evidence for a dietary role - mirroring patterns observed in more westerly areas of Central Asia^22,67^. Extant faunal assemblages from the small handful of documented domestic contexts in Mongolia indicate that horses became a key component of diet sometime during the Bronze Age^39,71^, a pattern mirrored in their frequency in ritual assemblages^33^. The lack of physical structures associated with later periods also implies a shift towards mostly ephemeral habitations and an increased level of mobility during this period^39^. While available samples are small (as dictated by the region’s fragmentary archaeological record) our data thus suggest an Early and Middle Bronze Age pastoral economy that differed markedly from the specialized, horse-focused pastoralism that characterizes later periods– in which these animals played a crucial role in diet, movement, and culture.

**Figure 8:**
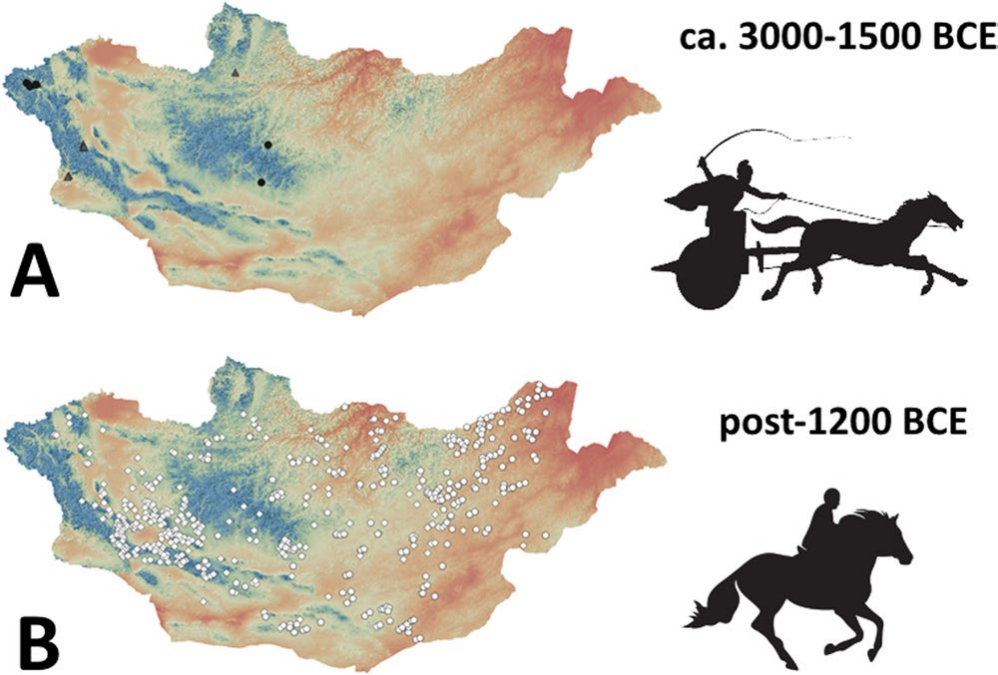
(**A**) Early and Middle Bronze Age archaeological sites (Afanasievo Chemurchek, and Munkhkhairkhan, ca. 3000–1500 BCE) and (**B**) Post-1200 BCE Late Bronze and Early Iron Age archaeological sites (DSKC and Slab Burial cultures) plotted against digital elevation model of Mongolia, with low elevation of 500 m shown in red, and high elevation (2500 + m) shown in blue. Data from Eregzen (2016). Icons in upper right show hypothesized horse transport technology in use during each period.

Changes in the location of ritual structures, monuments, and burials across the Bronze and Early Iron Ages imply a dramatic increase in the exploitation of dry, intermontane and open steppe regions during the second millennium BCE that may reflect changes in mobility associated with horse riding (Fig. 8). The Early and Middle Bronze Age were particularly dry in many areas of central and western Mongolia after 3000 BCE^72–74^, which may have driven down wild game abundance and made pastoral subsistence more attractive. With comparatively higher rainfall and seasonally productive food and water sources at high altitude, the high mountainous regions of Mongolia would have been the most stable and viable regions for pastoral herding – particularly if mobility was comparatively limited by the lack of mounted horseback riding. These mountain regions are the primary locations for chariot petroglyphs in Mongolia, which are not typically found in the country’s eastern reaches^75^. Although other non-cultural explanations such as the availability of suitable geology for rock art preservation in the Altai region could also explain this pattern, a shift towards widespread occupation of intermontane regions may be plausibly associated with the increased mobility associated with mounted horseback riding. The apparent fluorescence of pastoral occupation of steppe and steppe-desert regions may also reflect the unique ecological benefits of dry steppe associated with horse herding, including increased herd yields and improved health for animals moved over long distances in these areas^17^.

**Figure 9:**
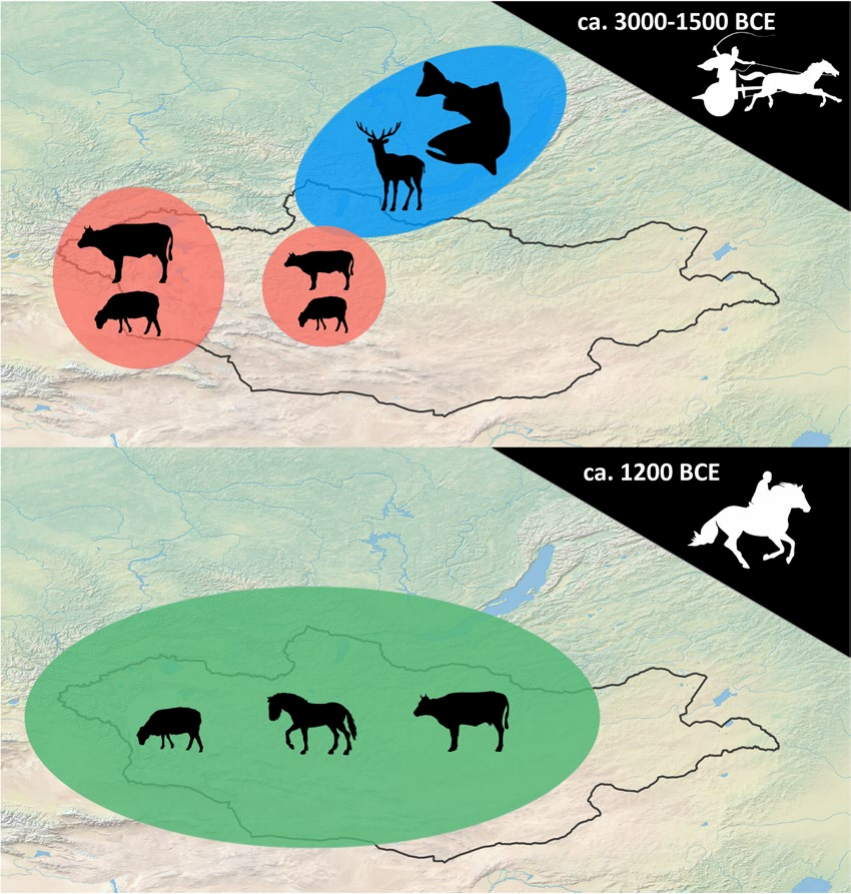
Idealized model for the impact of horse transport on pastoral lifeways in Mongolia during the late Bronze Age. Blue denotes hunting and gathering subsistence, red denotes low-mobility pastoral subsistence, and green denotes horse-based, high mobility pastoral subsistence. The top figure shows the clustering of pastoral lifeways in montane regions of western and central Mongolia during early and middle Bronze Age, and the bottom figure shows the widespread geographic distribution of Late Bronze Age and Early Iron Age pastoral sites, concurrent with both early evidence for horseback riding as well as increased economic exploitation of horses.

Our data – the first habitation sites of their kind with detailed taxonomic identifications from the early Bronze Age of Mongolia – support inferences from meta-analyses indicating that while pastoralism was practiced in parts of Central Asia and Mongolia by the early Bronze Age, the adoption of horse riding prompted dramatic alterations to the ecology of herding economies during the end of the second millennium BCE. Considering the distribution of documented monument sites across the Bronze Age, we hypothesize that the innovation or local adoption of horseback riding prompted a shift from localized transhumant exploitation of montane zones, with economies perhaps focused on sheep and cattle, to a more diverse pastoral economy where horses played a crucial role as livestock (Fig. 9). This pattern of horse-based subsistence and high residential mobility persisted from the DSK period through the time of the Mongol Empire^76^ and up to the present day^77^.

### Horses and herding transformations across central asia

The most compelling support for a transformation in the role of domestic horses observed in the Mongolian archaeological record at the end of the second millennium BCE comes from contemporaneous patterns in published archaeofaunal assemblages from western regions of Central Asia – indicating that this shift was both real and broad geographic significance. The available data support the idea that the incorporation of horses into chariot technology prompted the initial integration of domestic horses into pastoral lifeways for many Bronze Age Central Asian herders, as implied by Kohl^28^ and others. Horses and their secondary products (as suggested through lipid analysis of milk in ceramics) were likely still used in ritual activity during the early second millennium BCE, but they declined in general visibility in archaeological assemblages during this time^22^, and likely served a limited economic or dietary role^66^. In contrast, and as noted earlier by scholars working in areas such as SE Kazakhstan (e.g.^6,67^) a recognizable pattern of increase in horse remains at many sites is visible at the end of the second millennium BCE across a broad geographic region.

Some researchers (e.g.^11^) have rightly highlighted the potential discrepancy between the number of horse bones recovered from a given archaeological assemblage, and actual frequency of animals kept by a given group. In many contexts, riding horses have been kept in large numbers without leaving a corresponding zooarchaeological signature. Therefore, the frequency of horse bones found at any given site is, on its own, a poor reflection of the extant horse population associated with the site’s inhabitants. Nonetheless, our data indicate that at the end of the second millennium BCE, some areas witnessed saw *dietary* exploitation of domestic horses to a previously unforeseen extent, and that all study regions saw a general trend towards higher frequencies of horse bones in zooarchaeological assemblages. Acknowledging that %NISP is an imperfect measure, we argue that this pattern – increased dietary reliance on horses at many locations across the Eurasian Steppe – reflects the ability to control larger herds of horses initiated by the innovation of mounted horseback riding. In some cases, the ability to control more horses appears to have prompted an increased dietary reliance on horses as a source of meat – causing an increase in the frequency of horses in many archaeofaunal assemblages. It must be noted that broad-scale environmental differences between regions^6^, as well as cultural and microenvironmental factors^7^, are important influences on the composition of Central Asian herds and archaeofaunal assemblages. Compellingly, however, the pattern of increased frequency of archaeological horse remains during the late second millennium BCE can still be observed *within* various regions - including in the Trans-Ural region, the Central Steppes of Kazakhstan, and the arid desert-steppes of southern Central Asia (Fig. 7).

Together, this broad summary of extant scholarship supported by new data from Mongolia point to a pronounced increase in the numbers of horses used by pastoralists across multiple different ecozones of Central Asia at the end of the second millennium BCE. Aligned with a growing body of scholarship linking this period with the adoption of riding both in Mongolia^35–37,75^ and more broadly across Central Asia^25,28,67^, our results support a direct link between increased economic reliance on domestic horses and the emergence of mounted riding among East or Central Asian pastoralists – ca. 1200 BCE. This proposed link between riding and economic exploitation of horses helps to explain the limited economic and mortuary presence of horses noted in the Central Steppe^22,78^ and corroborated from our initial results in Mongolia.

### Implications: bronze age mongolia and LBA dispersals

A Late Bronze Age origin for mounted horseback riding may explain very important, broad-scale cultural and biological patterns across Inner Asia. Recent genomic sequencing efforts place the DSK horse as one of the most basal lineages among ancient domestic horses studied to date^21^. Spatial analysis of modeled radiocarbon dates indicates a rapid geographic expansion of horse sacrifice at DSK monuments across the Eastern Steppe^33^, concurrent with some of the oldest direct evidence for mounted horseback riding (equine osteological changes) from these same DSK horses^36^. Although these data come primarily from ritual contexts, our new data from early Bronze Age campsites seem to show a similar pattern mirrored in dietary assemblages. Deer Stones are recognized as the earliest clear progenitor for ‘animal-style’ art, a style that spread across most of Central Asia during the first millennium BCE^32,79^. Recent large-scale genomic research suggests that the expansion of DSK culture and later diffusion of animal art style co-occurred with westward gene flow into western Eurasia from proto-“Scythian” peoples in eastern Inner Asia during the first millennium BCE^80^. The innovation of mounted riding in Central or East Asia, and subsequent outward dispersal of human groups provides one compelling hypothesis to explain both westward gene flow and cultural transmission of animal style. In fact, recent careful experimental study of the use-wear patterns on second millennium BCE horse equipment from Central Asia points to the latter half of the second millennium BCE as the earliest possible date for widespread riding^26^. Recent human genomic data from DSK populations in northern Mongolian link these groups with ancestral Northeast Asian/Siberian hunter-gatherers^81^. The outward dispersal of these groups into westerly areas of Eurasia during the LBA, linked with the innovation or adoption of mounted horseback riding could thus explain the mixed pastoralist/hunter gatherer signal evidenced in many early Iron Age Saka/Scythian groups^80^.

## Conclusion

**Figure 10:**
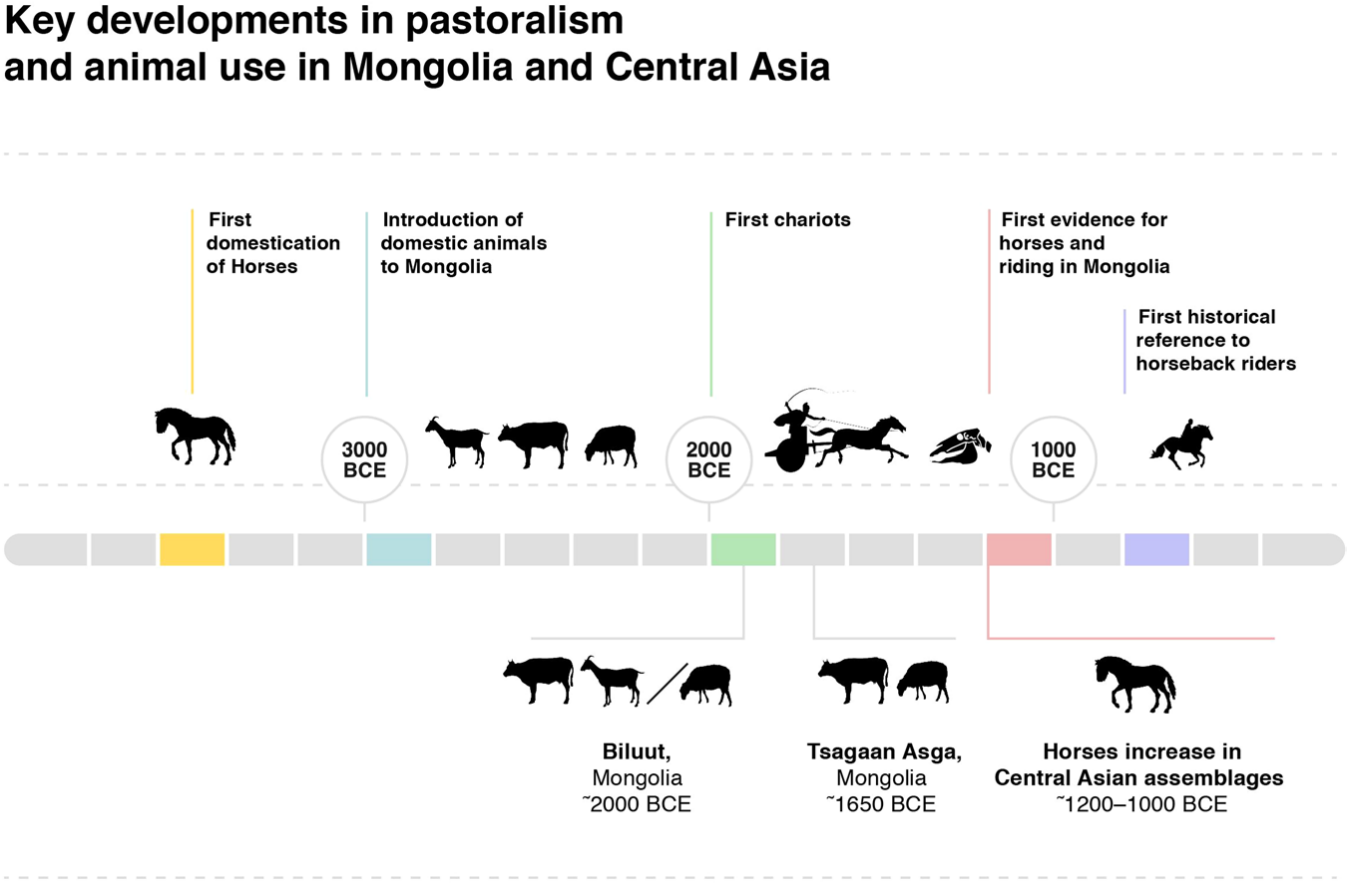
Timeline for horse domestication and key events related to early pastoralism in Mongolia and surrounding regions.

With links emerging between the emergence of horse transport and major changes to the structure of Bronze Age pastoral and hunter-gatherer economies in the Eastern Steppes of Eurasia, the broad-scale changes in the frequency of horses in faunal assemblages in western Central Asia appear to be an ecological response to innovations in horse transport. Faunal data show clearly that horses played a comparatively limited role in Early and Middle Bronze Age economies across much of Central Asia, patterns that appear replicated in the first direct insights into coeval Mongolian economies. Excavations at Tsagaan Asga and reanalysis of the assemblage from Biluut demonstrate a Middle Bronze Age pastoral economy in western Mongolia reliant on sheep and to a lesser extent, cattle, and utilizing permanent structures with large stone foundations – despite archaeological evidence that domestic horses were both known to Mongolian herders and present in ritual archaeological sites at a low level during this time. In contrast, horses flourished during the late Bronze Age, taking on both an important role in the pastoral diet and economy as well as markedly increased visibility in ritual contexts. Although the absolute frequency of horses varies widely across regions, cultures, and subsistence strategies, the internal consistency of the pattern of increased horse remains within each region of Central Asia is suggestive of a greater number of these animals on the landscape and a more prominent role for them in pastoral economies. We summarize new and extant data relevant to key stages of domestic animal use in Mongolia in Fig. 10.

Supporting the multi-stage chronology for equine transport proposed by earlier influential scholars^22,28^ but contra to Anthony^1^ and others, we propose that this revolutionary transition is best explained by a second millennium BCE innovation of mounted horseback riding from earlier use in traction. Mounted riding would have increased the utility of horses as a transport animal, thereby encouraging groups to maintain greater numbers of horses even in a subsistence framework that otherwise placed little emphasis on them. Although small numbers of horses may have been tied, hobbled, or corralled for use in chariot teams or secondary products, large herds cannot be easily tended without effective transportation. The ability to ride horses would have dramatically increased the range of viable economic uses for horses by human societies in Inner Asia - perhaps allowing horses to be managed in meaningful numbers on a free range for the first time. In Mongolia, this likely bolstered the value of horses as a source of meat and dairy products, and enabled herders to effectively utilize the dry low-elevation areas of the Eastern Steppe for the first time. Although pastoralism was apparently practiced in Mongolia from ca. 3000 BCE, it appears that only after the innovation of mounted riding, associated changes to the ecological parameters of pastoralism, and the fluorescence of horse-based nomadic culture in Mongolia were hunting and gathering displaced as the dominant economic strategy in some northern regions. Innovations in horse transport – first the chariot, followed by mounted horseback riding – may have stimulated widespread transformations in Bronze Age pastoral economies, and help explain large scale population dispersals between east and west across Eurasia.

## Supplementary information

Supplementary Information.
