# Author Correction: Early Pastoral Economies and Herding Transitions in Eastern Eurasia

**DOI:** 10.1038/s41598-020-60516-2

**Published:** 2020-03-06

**Authors:** William Timothy Treal Taylor, Julia Clark, Jamsranjav Bayarsaikhan, Tumurbaatar Tuvshinjargal, Jessica Thompson Jobe, William Fitzhugh, Richard Kortum, Robert N. Spengler, Svetlana Shnaider, Frederik Valeur Seersholm, Isaac Hart, Nicholas Case, Shevan Wilkin, Jessica Hendy, Ulrike Thuering, Bryan Miller, Alicia R. Ventresca Miller, Andrea Picin, Nils Vanwezer, Franziska Irmer, Samantha Brown, Aida Abdykanova, Daniel R. Shultz, Victoria Pham, Michael Bunce, Katerina Douka, Emily Lena Jones, Nicole Boivin

**Affiliations:** 1 CU Museum of Natural History/Department of Anthropology, CU 218, Boulder, CO 80309 USA; 2 Department of Archaeology, Max Planck Institute for the Science of Human History, 10 Kahlaische Str., Jena, 07745 Germany; 3 Flinders University, Australia, Sturt Road, Bedford Park, South Australia 5042 Australia; 4 National Museum of Mongolia, Juulchiny Str-1, Ulaanbaatar, 21046 Mongolia; 5 Graduate School of Human Development in Landscapes, University of Kiel, Johanna-Mestorf Str 2-6, R.156, D - 24118 Kiel, Germany; 6 Department of Geology and Geological Engineering, Colorado School of Mines, 1500 Illinois St., Golden, CO 80401 USA; 7 Arctic Studies Center, Smithsonian National Museum of Natural History, Washington, D.C. 20560 USA; 8 Department of Philosophy and Humanities, East Tennessee State University, 276 Gilbreath Dr, Johnson City, TN 37614 USA; 9 Institute of Archaeology and Ethnography, Siberian Branch Russian Academy of Science, 17 Lavrentieva Avenue, Novosibirsk, 630090 Russia; 10 Novosibirsk State University, 1, Pirogova Str., Novosibirsk, Russia; 11 Trace and Environmental DNA (TrEnD) Laboratory, Curtin University, Kent Street, Bentley, WA 6102 Australia; 12 Department of Geography, University of Utah, 260 Central campus Drive Room 4625, Salt Lake City, UT 84112 USA; 13 Wyoming Geographic Information Science Center, Department of Geography, University of Wyoming, 1000 E. University Ave, Laramie, WY 82071 USA; 14 Faculty of History, University of Oxford, George Street, OX1 2RL Oxford, UK; 15 Anthropology Program, American University of Central Asia, Aaly Tokombaev st. 7/6, 720060 Bishkek, Kyrgyzstan; 16 Departments of Anthropology and History, McGill University, 855 Sherbrooke Street West, Montreal, Quebec H3A 2T7 Canada; 17 University of Sydney, Australia, Camperdown, NSW 2006 Australia; 18 Department of Anthropology, University of New Mexico, MSC01-1040, Albuquerque, NM 87131 USA; 19 Department of Anthropology, Museum of Anthropological Archaeology, University of Michigan, Ann Arbor, MI 48109 USA

Correction to: *Scientific Reports* 10.1038/s41598-020-57735-y, published online 22 January 2020

This Article contains a typographical error in the Introduction section under subheading ‘Understanding Early Horse Domestication and Transport’ where,

“Historical records refer to horse-mounted warriors in western Asia by the 8th century BCE, while archaeological finds from localities like Arzhan 2 in southern Tuva show specialized horse equipment (bronze snaffle bits) and equine vertebral pathologies linked with mounted riding in Central Asia by the late 9th century BCE^31^.”

should read:

“Historical records refer to horse-mounted warriors in western Asia by the 8th century BCE, while archaeological finds from localities like Arzhan in southern Tuva show specialized horse equipment (bronze snaffle bits) and equine vertebral pathologies linked with mounted riding in Central Asia by the late 9th century BCE^31^.”

