# Supplementary material for: Early Pastoral Economies and Herding Transitions in Eastern Eurasia
Source: Sci Rep. 2020 Jan 22;10:1001. doi: 10.1038/s41598-020-57735-y (PMC6976682; doi:10.1038/s41598-020-57735-y)
Supplement: Supplementary file 1 — Supplementary Information. [file 41598_2020_57735_MOESM1_ESM.pdf]

Early Pastoral Economies and Herding Transitions in Eastern Eurasia

William Timothy Treal Taylor1,2* Julia Clark3 Jamsranjav Bayarsaikhan4, Tumurbaatar Tuvshinjargal5, Jessica Thompson Jobe6, William Fitzhugh7, Richard Kortum8, Robert N. Spengler III2, Svetlana Shnaider9,10, Frederik Valeur Seersholm11, Isaac Hart12, Nicholas Case13, Shevan Wilkin2, Jessica Hendy2, Ulrike Thuering2, Bryan Miller 2,14, Alicia R. Ventresca Miller2,Andrea Picin2, Nils Vanwezer2, Franziska Irmer2, Samantha Brown2, Aida Abdykanova15, Daniel R. Shultz16, Victoria Pham17,Michael Bunce11, Katerina Douka2,Emily Lena Jones18, Nicole Boivin2

*Correspondence to william.taylor@colorado.edu

1 CU Museum of Natural History/Department of Anthropology, (CU 218, Boulder, CO, 80309, william.taylor@colorado.edu)

2 Department of Archaeology, Max Planck Institute for the Science of Human History (10 Kahlaische Str. Jena, Germany 07745)

3 Flinders University, Australia (Sturt Road, Bedford Park, South Australia, Australia 5042, julia.clark@flinders.edu.au)

4 National Museum of Mongolia (Juulchiny Str-1, Ulaanbaatar-21046, Mongolia jabayarsaikhan@gmail.com)

5 Graduate School of Human Development in Landscapes, University of Kiel (Johanna-Mestorf Str 2-6, R.156, D - 24118 Kiel, Germany, ttumurbaatar@gshdl.uni-kiel.de)

6 Department of Geology and Geological Engineering, Colorado School of Mines (1500 Illinois St., Golden, CO, 80401, USA, jtjobe@mines.edu)

7 Arctic Studies Center, Smithsonian National Museum of Natural History (Washington, D.C. USA 20560, fitzhugh@si.edu)

8 Department of Philosophy and Humanities, East Tennessee State University (276 Gilbreath Dr, Johnson City, TN, USA 37614, kortumr@mail.etsu.edu)

9 Institute of Archaeology and Ethnography, Siberian Branch Russian Academy of Science, (17 Lavrentieva Avenue, Novosibirsk, Russia, 630090)

10 Novosibirsk State University (1, Pirogova Str., Novosibirsk, Russia, sveta.shnayder@gmail.com)

11 Trace and Environmental DNA (TrEnD) Laboratory, Curtin University, Kent Street, Bentley, WA 6102, Australia (frederikseersholm@gmail.com)

12 Department of Geography, University of Utah (260 Central campus Drive Room 4625, Salt Lake City, UT, 84112, i.hart@utah.edu)

13 Wyoming Geographic Information Science Center, Department of Geography, University of Wyoming, (1000 E. University Ave. Laramie, WY 82071, ncase2@uwyo.edu)

14 Faculty of History, University of Oxford (George Street, OX1 2RL Oxford, UK, bryan.miller@history.ox.ac.uk)

15 Anthropology Program, American University of Central Asia (Aaly Tokombaev st. 7/6, Kyrgyzstan 720060, abdykanova_a@auca.kg)

16 Departments of Anthropology and History, McGill University (855 Sherbrooke Street West, Montreal, Quebec, Canada H3A 2T7, daniel.shultz@mail.mcgill.ca)

17 University of Sydney, Australia (Camperdown NSW 2006, Australia, vtra6880@uni.sydney.edu.au)

18 Department of Anthropology, University of New Mexico (MSC01-1040, Albuquerque, NM, 87131, USA, elj@unm.edu)

Supplementary information

Section S1. ZooMS analysis

Technical details for MALDI-TOF ZooMS analysis.

Reflector 3.0x 1917V
Smartbeam Parameter Set 4_large, frequency 2000Hz
Sample Rate 5.00GS/s
Realtime smoothing Off
Baseline Offset Adjustment 0.0%
Analog offset 1.2mV

Matrix suppression: Deflection up to 450 Da
Processing Method SC_Peptide_Cent

Ion Source 1: 18.88kV
Ion Source 2: 16.53 kV
Lens: 8.52 kV
Reflector: 21 kV
Reflector 2: 9.6 kV

Laser: Global Attenuator Offset 25%
Digitizer sensitivity: 100mV
Trigger level: 800mV

Detector Gain Voltages: Linear base 2500V, Reflector Base 1700V, Reflector Boost 50V

Table S1. Diagnostic peptide markers and taxonomic identifications by specimen.

Specimen ID	Artifact #	Site	Context	Element	Taphonomic notes	Taxonomic ID (Morph.)	Taxonomic ID (ZooMS)	P1	A	A'	B	C	P2	D	E	F	F'	G	G'
S001	SOY-100232	Bagsagiin Bulan	O9, Cut 1	Indet.	Digestion?	Indet.	Cervid/Saiga/Ovinae	1105.6	-	1196.6	1427.7	-	1648.8	2131.1	2792.3	2883.4	2899.4	3017.5	3033.5
S002	SOY-100243	Bagsagiin Bulan	O8, Dep 1	Indet.	Burned (calcined)	Indet.	Indeterminate	1105.6	-	-	1427.7	-	-	-	-	2883.4	-	-	-
S003	SOY-100163	Bagsagiin Bulan	M8, Dep 1	Indet.	Root etching	Indet.	Ovis	1105.6	1180.6	1196.6	1427.7	1580.8	1648.8	2131.1	2792.3	2883.4	2899.4	3017.5	3033.5
S004	SOY-100237	Bagsagiin Bulan	M/N9, Dep 1 (under rock)	Cervical vertebra (C6?)		Rodentia	Castor	1105.6	-	-	1427.7	-	-	2129.7	-	2883.4	2899.4	-	-
S005	SOY-100098	Bagsagiin Bulan	O8, Dep 0	Indet.	Burned (carbonized)	Indet.	Cervid/Saiga/Ovinae	1105.6	-	-	1427.7	-	1648.8	-	-	2883.4	2899.4	3017.5	3033.5
S006	SOY-100212	Bagsagiin Bulan	Q9, Dep 1	Indet.		Indet.	Capreolus sp.	1105.6	1180.6	-	1427.7	-	1648.8	2131.1	2792.3	2883.4	2899.4	3043.4	3059.4
S007	SOY-100231	Bagsagiin Bulan	O9, Cut 1	Indet.		Indet.	Cervid/Saiga/Ovinae	1105.6	1180.6	-	1427.7	-	-	2131.1	2792.3	2883.4	-	3017.5	3033.5
S009A	SOY-100192	Bagsagiin Bulan	M8, Dep 1	Maxilla fragment (L)	Trampling	Rodentia	Unknown Rodentia	1105.6	1182.6	-	1453.7	-	-	2159.1	-	2853.4	2899.4	2983.5	2999.5
S009B	SOY-100192	Bagsagiin Bulan	M8, Dep 1	Indet.	Trampling	Indet.	Ovis	1105.6	-	1196.6	1427.7	1580.8	1648.8	2131.1	2792.3	2883.4	2899.4	3017.5	3033.5
S010A	SOY-100194	Bagsagiin Bulan	P9, Dep 0	Indet.	Burned (carbonized, calcined), root etching	Indet.	Indeterminate	-	-	-	-	-	-	-	-	-	-	-	-
S010B	SOY-100194	Bagsagiin Bulan	P9, Dep 0	Indet. (long bone)	Burned (carbonized), spiral fracture, root etching	Indet.	Cervidae/Bovidae	-	-	-	1427.7	-	-	2131.1	-	2883.4	-	-	-
S011	SOY-100113	Bagsagiin Bulan	M8, Dep 1	Mandible (articular surface fragment, L)	Cut mark	Ruminant	Deer/Gazelle/Saiga	1105.6	1180.6	1196.6	1427.7	1550.8	1649.8	2131.1	2792.3	2883.4	2899.4	3017.5	3033.5
T001	TSA-100090	Tsagaan Asga	K2, Dep 1/2 (contact)	Indet.	Burned (carbonized)	NA	Ovis	1105.6	1180.6	1196.6	1427.7	1580.8	1648.8	2131.1	2792.3	2883.4	2899.4	3017.5	3033.5
T002	TSA-100056	Tsagaan Asga	J4, Deposit 2	Indet.	Spiral fracture	Indet.	Ovis	1105.6	-	-	1427.7	1580.8	1648.8	2131.1	2792.3	2883.4	2899.4	3017.5	3033.5
T003A	TSA-100098	Tsagaan Asga	Uncertain (screen), Deposit 2	Indet. (long bone)	Spiral fracture, digestion?	Ovis/Capra	Ovis	1105.6	1180.6	1196.6	1427.7	1580.8	1648.8	2131.1	2792.3	2883.4	-	3017.5	3033.5
T003B	TSA-100098	Tsagaan Asga	Uncertain (screen), Deposit 2	Indet.	Burned (carbonized)	Indet.	Cervid/Saiga/Ovinae	1105.6	1180.6	1196.6	1427.7	-	1648.8	-	2792.3	2883.4	-	3017.5	3033.5
T003C	TSA-100098	Tsagaan Asga	Uncertain (screen), Deposit 2	Indet.	Burned (carbonized)	Indet.	Cervid/Saiga/Ovinae	1105.6	1180.6	-	1427.7	-	1648.8	2131.1	2792.3	2883.4	-	3017.5	3033.5

T003D	TSA-100098	Tsagaan Asga	Uncertain (screen), Deposit 2	Indet.	Burned (carbonized)	Indet.	Ovis	1105.6	-	-	-	1580.8	1648.8	-	2792.3	2883.4	2899.4	3017.5	3033.5
T003E	TSA-100098	Tsagaan Asga	Uncertain (screen), Deposit 2	Indet.	Burned (carbonized)	Indet.	Cervid/Saiga/Ovinae	1105.6	-	-	-	-	1648.8	-	2792.3	2883.4	-	3017.5	3033.5
T004	TSA-100092	Tsagaan Asga	K2, Deposit 2	Long bone	Spiral fracture, root etching	Ovis/Capra	Cervid/Saiga/Ovinae	1105.6	1180.6	1196.6	1427.7	-	1648.8	2131.1	2792.3	2883.4	2899.4	3017.5	3033.5
T005	TSA-100082	Tsagaan Asga	K2, Dep 1/2 (contact)	Indet.	Trampling	Indet.	Ovis	1105.6	-	1196.6	1427.7	1580.8	1648.8	2131.1	2792.3	2883.4	2899.4	3017.5	3033.5
T006	TSA-100092	Tsagaan Asga	K3, Dep 1/2 (contact)	Indet.	Burned (carbonized, calcined)	Indet.	Bos	1105.6	1192.7	1208.8	1427.7	1580.8	1648.8	2131.1	2792.3	2853.4	-	3017.5	3033.5
T007	TSA-100081	Tsagaan Asga	J3, Dep 1/2 (contact)	Indet.		Indet.	Indet.	1105.6	-	-	-	-	1648.8	-	-	-	-	3017.5	3033.5
T008	TSA-100028	Tsagaan Asga	I3, Deposit 1	Indet.	Burned (calcined), root etching	Indet.	Bos	1105.6	1192.7	1208.8	1427.7	-	1648.8	2131.1	-	2853.4	-	3017.5	3033.5
T009	TSA-100083	Tsagaan Asga	K2, Dep 1/2 (contact)	Indet.	Burned (calcined), spiral fracture	Indet.	Ovis	1105.6	1180.6	-	1427.7	1580.8	1648.8	2131.1	2792.3	2883.4	2899.4	3017.5	3033.5
T010	TSA-100065	Tsagaan Asga	J2, Dep 1/2 (contact)	Indet.	Spiral fracture, root etching	Indet.	Cervidae/Bovidae	1105.6	-	-	-	-	-	2131.1	-	-	-	3017.5	3033.5
T011	TSA-100057	Tsagaan Asga	J4, Deposit 2	Indet.	Burned (calcined), spiral fracture, cut mark	Indet.	Ovis	1105.6	1180.6	-	1427.7	1580.8	1648.8	2131.1	2792.3	2883.4	2899.4	3017.5	3033.5
T012A	TSA-100074	Tsagaan Asga	K2, Dep 1/2 (contact)	Indet.	Spiral fracture	Indet.	Cervid/Saiga/Ovinae	1105.6	-	-	1427.7	-	1648.8	2131.1	2792.3	2883.4	-	3017.5	3033.5
T012B	TSA-100074	Tsagaan Asga	K2, Dep 1/2 (contact)	Indet.		Indet.	Cervid/Saiga/Ovinae	1105.6	-	-	1427.7	-	1648.8	2131.1	2792.3	2883.4	-	3017.5	3033.5
T012C	TSA-100074	Tsagaan Asga	K2, Dep 1/2 (contact)	Indet.	Spiral fracture	Indet.	Cervid/Saiga/Ovinae	1105.6	1180.6	-	1427.7	-	1648.8	2131.1	2792.3	2883.4	-	3017.5	3033.5
T013	TSA-100088	Tsagaan Asga	K2, Dep 1/2 (contact)	Indet.	Burned (calcined)	Indet.	Cervid/Saiga/Ovinae	1105.6	-	-	1427.7	-	1648.8	-	2792.3	2883.4	-	3017.5	-
T014	TSA-100101	Tsagaan Asga	K3, Dep 1/2 (contact)	Astragalus?	Digestion or use wear?	Large mammal	Cervidae/Bovidae	1105.6	-	-	-	1580.8	1648.8	2131.1	2792.3	-	-	3017.5	3033.5
T015	TSA-100100	Tsagaan Asga	Uncertain, Deposit 1	Indet.		Indet.	Cervidae/Bovidae	1105.6	-	-	-	-	1648.8	-	2792.3	-	-	3017.5	3033.5
T016	TSA-100023	Tsagaan Asga	K2, Deposit 1	Indet.	Burned (calcined)	Indet.	Ovis	1105.6	-	-	1427.7	1580.8	1648.8	2131.1	2792.3	2883.4	2899.4	3017.5	3033.5
T017	TSA-100053	Tsagaan Asga	J3, Deposit 1	Indet.	Burned (calcined)	Indet.	Ovis	1105.6	-	-	1427.7	1580.8	1648.8	2131.1	2792.3	2883.4	2899.4	3017.5	-

T018	TSA-100089	Tsagaan Asga	K2, Dep 1/2 (contact)	Indet. (long bone)	Root etching	Long bone fragment	Ovis	1105.6	-	-	1427.7	1580.8	1648.8	2131.1	2792.3	2883.4	-	3017.5	-
T019A	TSA-100071	Tsagaan Asga	J5, Deposit 2	Indet.	Burned (calcined)	Indet.	Cervid/Saiga/Ovinae	1105.6	1180.6	-	1427.7	-	1648.8	2131.1	2792.3	2883.4	2899.4	3017.5	3033.5
T019C	TSA-100071	Tsagaan Asga	J5, Deposit 2	Indet.	Burned (calcined)	Indet.	Bos	1105.6	-	-	1427.7	-	-	-	-	2853.4	-	-	-
T020A	TSA-100027	Tsagaan Asga	K2, Deposit 1	Indet. (long bone)	Trampling	Indet.	Cervid/Saiga/Ovinae	1105.6	-	-	1427.7	-	1648.8	-	2792.3	2883.4	2899.4	3017.5	-
T020B	TSA-100027	Tsagaan Asga	K2, Deposit 1	Indet. (long bone)	Trampling	Indet.	Cervid/Saiga/Ovinae	1105.6	-	1196.6	1427.7	-	1648.8	2131.1	2792.3	-	-	3017.5	-
T020C	TSA-100027	Tsagaan Asga	K2, Deposit 1	Indet.		Indet.	Cervid/Saiga/Ovinae	1105.6	-	-	1427.7	-	1648.8	-	2792.3	2883.4	-	3017.5	-
T021A	TSA-100072	Tsagaan Asga	K4, Dep 1/2 (contact)	Indet.		Indet.	Cervidae/Bovidae	1105.6			1427.7	1580.8	1648.8	-	-	-	-	-	-
T021B	TSA-100072	Tsagaan Asga	K4, Dep 1/2 (contact)	Tooth enamel	Digestion?	Ovis/Capra	Ovis	1105.6	-	-	1427.7	1580.8	1648.8	2131.1	2792.3	2883.4	-	3017.5	-
T021C	TSA-100072	Tsagaan Asga	K4, Dep 1/2 (contact)	Tooth enamel		Ovis/Capra	Ovis	1105.6	-	-	1427.7	1580.8	1648.8	2131.1	2792.3	2883.4	2899.4	3017.5	3033.5
T021D	TSA-100072	Tsagaan Asga	K4, Dep 1/2 (contact)	Indet.	Burned (calcined)	Indet.	Ovis	1105.6	1180.6	-	1427.7	1580.8	1648.8	2131.1	2792.3	2883.4	-	3017.5	-
T022	TSA-100086	Tsagaan Asga	K2, Dep 1/2 (contact)	Tooth (upper left M2/M3)	Root etching	Ovis/Capra	Ovis	1105.6	1180.6	1196.6	1427.7	1580.8	1648.8	2131.1	2792.3	2883.4	2899.4	3017.5	3033.5
T023	TSA-100012	Tsagaan Asga	I2, Deposit 1	Mandible (L)	Trampling	Arvicolinae	Unknown Rodentia	1105.6	-	-	1453.7	-	1609.8	-	-	2883.4	2899.4	-	3003.5
T024	TSA-100022	Tsagaan Asga	K2, Deposit 1	Indet.	Trampling, root etching	Indet.	Cervidae/Bovidae	1105.6	-	-	1427.7	-	1648.8	-	2792.3	2883.4	-	3017.5	-
T025A	TSA-100097	Tsagaan Asga	K3, Dep 1/2 (contact)	Indet.		Indet.	Cervid/Saiga/Ovinae	1105.6	-	-	1427.7	-	1648.8	2131.1	2792.3	-	2899.4	3017.5	3033.5
T025B	TSA-100097	Tsagaan Asga	K3, Dep 1/2 (contact)	Indet.		Indet.	Ovis	1105.6	1180.8	-	1427.7	1580.8	1648.8	-	2792.3	2883.4	-	3017.5	3033.5
T025C	TSA-100097	Tsagaan Asga	K3, Dep 1/2 (contact)	Indet.		Indet.	Bos	1105.6	-	-	1427.7	1580.8	1648.8	2131.1	2792.3	2853.4	-	3017.5	3033.5
T025D	TSA-100097	Tsagaan Asga	K3, Dep 1/2	Indet.	Root etching	Indet.	Ovis	1105.6	-	1196.6	1427.7	1580.8	1648.8	2131.1	2792.3	2883.4	2899.4	3017.5	3033.5

Section S2. Bagsagiin Bulan.

Section 2A. Geoarchaeology and Geomorphology of Bagsagiin Bulan

Jessica A. Thompson Jobe, Department of Geology and Geological Engineering, Colorado School of Mines

Geologic and Geomorphologic Setting of the Darkhad Basin

The Darkhad Basin, located in northern Mongolia, is an extensional basin at the western end of the Baikal Rift. The basin is ~100 km long and 20-40 km wide, and is covered by a number of small lakes. The basin is surrounded by mountains on all sides, with an active normal fault located along the eastern end of the basin (Bacon et al. 2003). The basin contains Pliocene through Holocene sediments (Spirkin 1970). The bedrock underlying the western part of the basin is limestone and dolomite of the Khesen Formation and granite and gneiss. The broad, low hills, including the Soyo Hill, are entirely bedded limestone.

Pleistocene-Holocene paleolakes filled the basin, created by glacial advance and damming of the Shishged Gol at its outlet at the northwestern end of the basin. The ice-dammed lakes are recorded by up to 47 shorelines preserved around the margins of the basin, with the highest shorelines created during MIS5b (Krivonogov et al. 2005) or early to middle MIS 3 (Gillespie et al. 2008). Previous studies have documented two widespread lacustrine terraces: 1) a basin-wide 8-15 m thick Upper Pleistocene terrace at ~1550 m; and 2) a locally distributed 15-20 m thick Upper Pliocene-Lower Pleistocene terrace at ~1560-1600 m (Krivonogov et al. 2012). Paleolake Darkhad experienced variable lake levels during the late Pleistocene through the Holocene (Krivonogov et al. 2012). It was minimal or disappeared between 12-9.6 ka and after 4.5-3.9 ka, was relatively deep from 9.6 to 7.1 and 6.4 to 4.5 ka, and was relatively shallow between 7.1 and 6.4 ka (Krivonogov et al. 2012).

At Hugiin Gol, the last maximum glacier deposited moraines that terminated at ~1672 m (Gillespie et al. 2008). The moraines created a post-glacial lake (Carson et al. 2003), which was breached after the last highstand (>1650 m) of Paleolake Darkhad. Three boulders from the distal end of the moraine were dated using cosmogenic ^{10}Be exposure dating, yielding ages of 38.6 +/- 2.0, 24.9, and 14.2 ka (Gillespie et al. 2008). The older moraine is regarded as having 10Be inheritance from previous exposure, and the moraine was likely deposited during MIS 2 (Gillespie et al. 2008). Three boulders from a left-lateral till terrace (kame) ranged in age from 95-207 ka, but were not corrected for erosion (Gillespie et al. 2008), and the scatter in ages may result from episodic burial and exhumation. A beach just east of modern-day Zayaday Nuur may have been the end of an arm of a 1602-m high stand of Paleolake Darkhad, and dates to ~11,000 cal BCE (Gillespie et al. 2008).

We focused on continuing the work of Putnam (2016) by examining the stratigraphy of fluvial terraces formed along the Hugiin Gol (Figure S2A.1). Over the course of 10 days, a total of 5 geological test pits were hand-dug into the T1 terrace surface along the southern bank of the Hugiin Gol near the archaeological site of Bagsagiin Bulan. The test pits were 50 cm^2.

We followed the USDA standards (Schoeneberger et al. 2012) when describing the soil within the geological test pits. Preliminary descriptions include: color (wet/dry), texture, structure, consistency, lower boundaries, rock fragments, and horizon designation (Birkeland 1999). All profiles were described, sketched, and photographed in the field. In addition, I collected ~200 g of bulk sediment from every 10 cm for soil descriptions and paleoenvironmental analysis.

Geomorphology Field Observations

There are 3 primary terrace levels at Bagsagiin Bulan (Fig. S2A.2). The highest, broadest terrace is the T3 glacial outwash plain. T2 is ~5-10 m below T3, and T1 is ~5-7 m below T2. All of the terraces have well rounded to subrounded granitic boulders, up to 2 m in diameter. The geological test pits excavated the uppermost stratigraphy of the T1 terrace level, and are described below in detail.

Although, none of these terraces are dated, the broader glacial outwash terrace (T3) likely formed during or immediately after the last glacial maximum (~14 ka) (Gillespie et al. 2008), but it could have been formed by earlier glaciations. Commonly, these types of terraces are formed when the base level (in this case, Tsagaan Nuur) changes in response to climate or tectonics. Using previous studies on Holocene changes in lake levels, we infer that T2 and T1 were formed ~7 and ~4.5 ka, respectively, when lake levels fell in the middle Holocene (Krivonogov et al. 2012).

Georchaeology Field Observations

We hand-dug 5 test pits at the Bagsagiin Bulan site to examine the stratigraphy.

The Bagsagiin Bulan site is located on the lowest terrace (T1) next to the river (Fig. SA2.2, Fig. S2A.3). The first 4 test pits were aligned approximately north-south beginning at from ~10 m from the riverbank, at 10 m intervals, onto the stable terrace surface. The last test pit (GTP-5) was located next to the archaeological excavation to corroborate the stratigraphy (Fig. S2A.5). At each pit, we excavated the sediment, described the soils/stratigraphy, and sampled each pit at ~10 cm intervals for paleoenvironmental and soil analysis (results pending). The observations and descriptions from the 5 test pits revealed several correlative subsurface layers.

At GTP-3 and GTP-4, the modern surface has a well-developed soil (Fig. S2A.4), with large fluvial boulders at depth. The boulders are primarily granite, and are subrounded with diameters of 10-40 cm. These boulders were likely originally part of the glacial features upstream (either in moraines or part of the glacial outwash plain) that were transported downstream by the river.

GTP-1 and GTP-2, closer to the river, had different, but correlative, subsurface stratigraphy (Fig. S2A.4). The base of both pits had well-rounded fluvial cobbles and boulders between 5-15 cm diameter, but as large as 30 cm in diameter. The clasts were composed of gneiss, quartzite, schist, granite, and undifferentiated metamorphic rocks, and many clasts had a thin, discontinuous carbonate coating. In GTP-1, fluvial and aeolian sands comprised the upper part of the stratigraphy, suggesting that erosion may have removed the stable surface seen in the

other test pits. In GTP-2, there was a possible paleosol at ~40 cm depth, covered by fluvial and aeolian sands that had an O (or OA) horizon soil surface. This same paleosol and overlying aeolian sands is also observed in GTP-5. The paleosol in GTP-5 is the level with cultural material.

In GTP-5, dark brown irregular mottling was observed on several of the pit walls. Commonly, darker brown or black mottles may represent an accumulation of organic matter. I interpret the mottles observed in GTP-5 to represent in situ decay of organic matter.

At Bagsagiin Bulan, the subsurface stratigraphy can be grouped into 3 generalized stratigraphic units, summarized below:

Unit 1: Unit 1 is present at the bottom of GTP-1 through GTP-4 pits, and is a fluvial (or glacio-fluvial) deposit with large, well-rounded granitic cobbles and boulders. The matrix is fine sand to sandy loam, with well-rounded grains. At the top of Unit 1 in GTP-2, there are lenses of gravel and pebbles in a coarse sand matrix, which may represent waning flow conditions during or following the deposition of the large boulders.

Unit 2: Unit 2 is the next deepest unit, overlying Unit 1 in an unconformable contact. Unit 2 is comprised of fluvial sands with uncommon well-rounded pebbles.

Unit 3: Unit 3 is the uppermost stratigraphic layer, and is found in GTP-1, -2, and -5 above a paleosol, which merges with the modern soil horizon away from the river to GTP-3 and GTP-4. Unit 3 is primarily aeolian sands that were blown in to the collapsed river bank where the site is located.

The Bagsagiin Bulan site appears to have been originally built on a stable terrace surface (preserved as the modern surface at GTP-3 and GTP-4, speculated to be ~4.5 ka or ca. 2500 BCE) near the river. Subsequent erosion of the river bend resulted in slumping of the surface closer to the river, resulting in the surface being preserved as a paleosol in GTP-2 and GTP-5 after a thin layer of younger aeolian sands (Unit 3) was later deposited on top. At GTP-1, the terrace surface (paleosol) was completely removed by erosion, and younger fluvial sands were deposited on top of Unit 1.

References

Bacon SN, Bayasgalan A, Gillespie A, Burke R (2003) Paleoseismic displacement measurements from landforms subjected to periglacial processes: observations along the Jarai Gol fault near the Tamyn Am Hills, Darhad Depression, northern Mongolia. XVI INQUA Congress Available at: https://gsa.confex.com/gsa/inqu/finalprogram/abstract_55972.htm.

Birkeland PW (1999) Soils and Geomorphology (Oxford University Press).

Carson RJ, Gillespie AR, Bayasgalan A (2003) Late Quaternary geology along the Högiin Gol, northern Mongolia. Proceedings of the Oregon Academy of Science 39:20–21.

Gillespie AR, Burke RM, Komatsu G, Bayasgalan A (2008) Late Pleistocene glaciers in Darhad Basin, northern Mongolia. Quat Res 69(2):169–187.

Ishikawa M, Yamkhin J (2016) Formation chronology of Arsain pingo, Darhad basin, northern Mongolia. Permafrost Periglacial Processes 27(3):297–306.

Krivonogov SK, Sheinkman VS, Mistruykov AA (2005) Stages in the development of the Darhad dammed lake (Northern Mongolia) during the Late Pleistocene and Holocene. Quat Int 136(1):83–94.

Krivonogov SK, et al. (2012) Solved and unsolved problems of sedimentation, glaciation and paleolakes of the Darhad Basin, Northern Mongolia. Quat Sci Rev 56:142–163.

Putnam, D., 2016. Preliminary Assessment of the Geomorphology and Geochronology of the Soyo Archaeological Site and its Environs, Khovsgol, Mongolia. In Northern Mongolia Archaeology Project: Soyo End of Year Field Report 2016. National Museum of Mongolia, Ulaanbaatar

Schoeneberger PJ, Wysocki DA, Benham EC Soil Survey Staff (2012) Field book for describing and sampling soils, ver. 3.0. Natural Resources Conservation Service, National Soil Survey Center, Lincoln, NE.

Spirkin, AI (1970) On ancient lakes of the Darkhat basin (Western Prikhubsugulye). Mesozoic and Cenozoic Geology of the West Mongolia, Nauka, Moscow, 143-150 (in Russian).

Figures

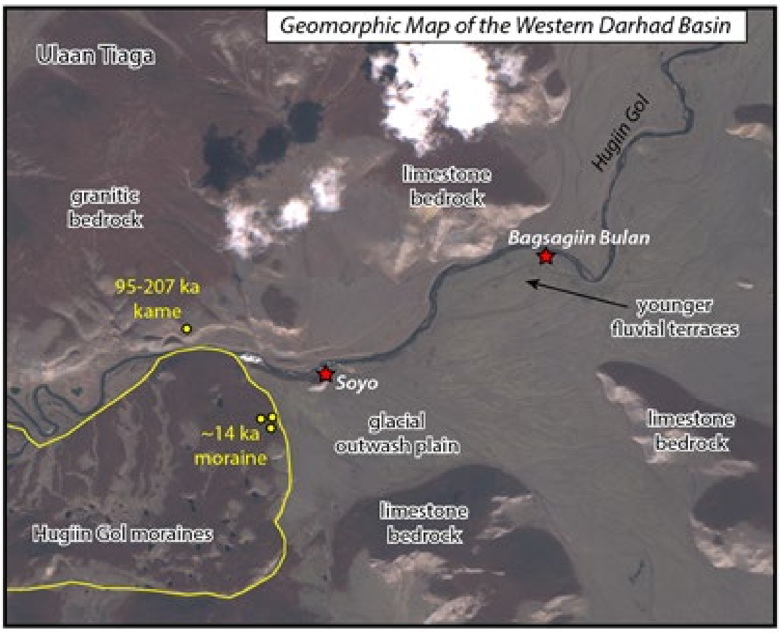

Fig. S2A.1. Geomorphic map of the western Darhad Basin, based on remote image interpretation and field observations. Base map is a satellite image is from Sentinel 2, L1C, produced by author Jessica Thompson Jobe using open-source and unrestricted, uncopyrighted data from the ESA Sentinel 2 (https://sentinel.esa.int/documents/247904/690755/Sentinel_Data_Legal_Notice). The two archaeological sites are shown as red stars. Previous geochronology on glacial features at Hugiin Gol (Gillespie et al., 2008) are marked by the yellow circles. Bedrock interpretations are based on field observations.

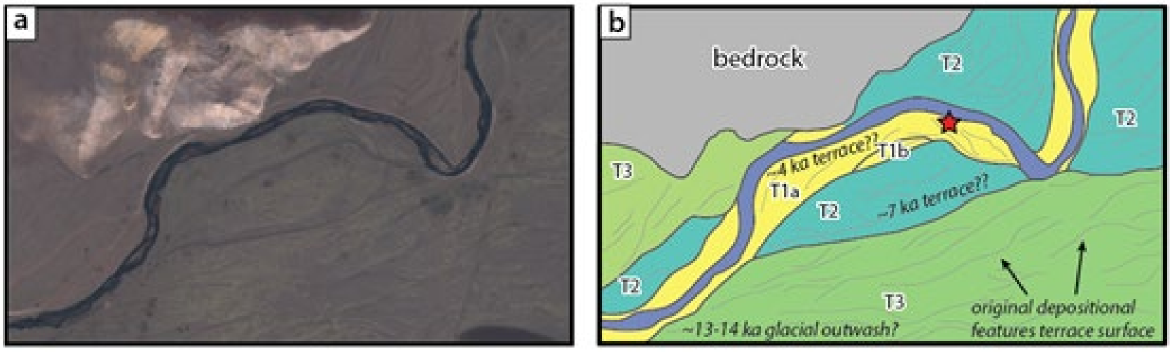

Fig. S2A.2. Geomorphic map of the area near the Bagsagiin Bulan archaeological site. (a) Satellite image and (b) geomorphic interpretation of the Bagsagiin Bulan site, marked by red star. View locations of photos in Fig. S2A.3 shown in (b). Base map is a satellite image is from Sentinel 2, L1C, produced by author Jessica Thompson Jobe using open-source and unrestricted, uncopyrighted data from the ESA Sentinel 2 (https://sentinel.esa.int/documents/247904/690755/Sentinel_Data_Legal_Notice).and mapping was created using QGIS v. 2.14.3 (available at

https://qgis.org/en/site/about/index.html). Ages for surfaces are interpreted and estimated based on downstream lake levels in Paleolake Darhad from Krivonogov et al., 2012 and glaciations from Gillespie et al., 2008.

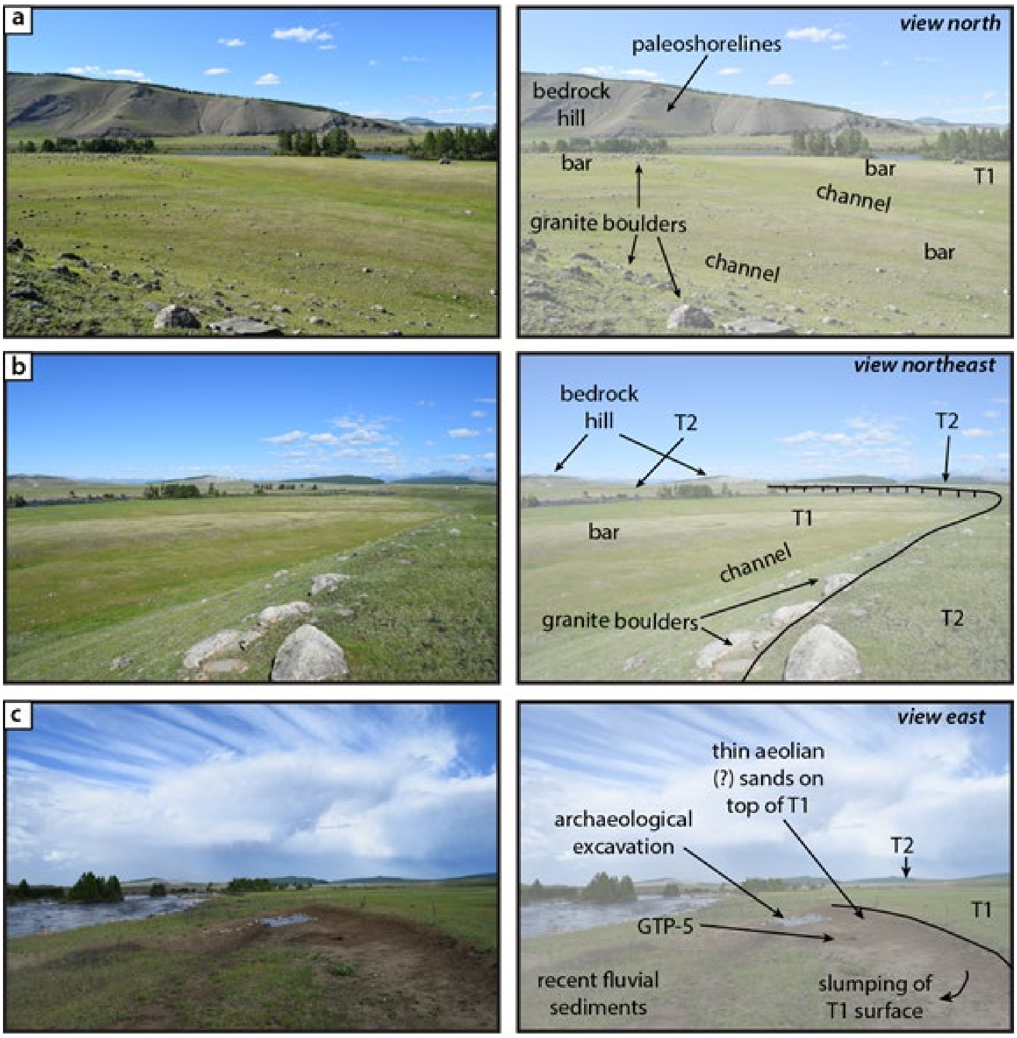

Fig. S2A.3. Field photos of the Bagsagiin Bulan archaeological site. (a) View to the north across T1. Original depositional features of the terrace are marked. Shorelines from Paleolake Darkhad are visible on bedrock in the distance. (b) View to the northeast from the top of T2, illustrating the typical surface characteristics of the terraces. (c) View to the east on T1, next to the archaeological excavation. Recent fluvial sediments were observed closer to the river, with thin aeolian sands deposited near the slumped surface and on top of T1. The location of GTP-5 is shown.

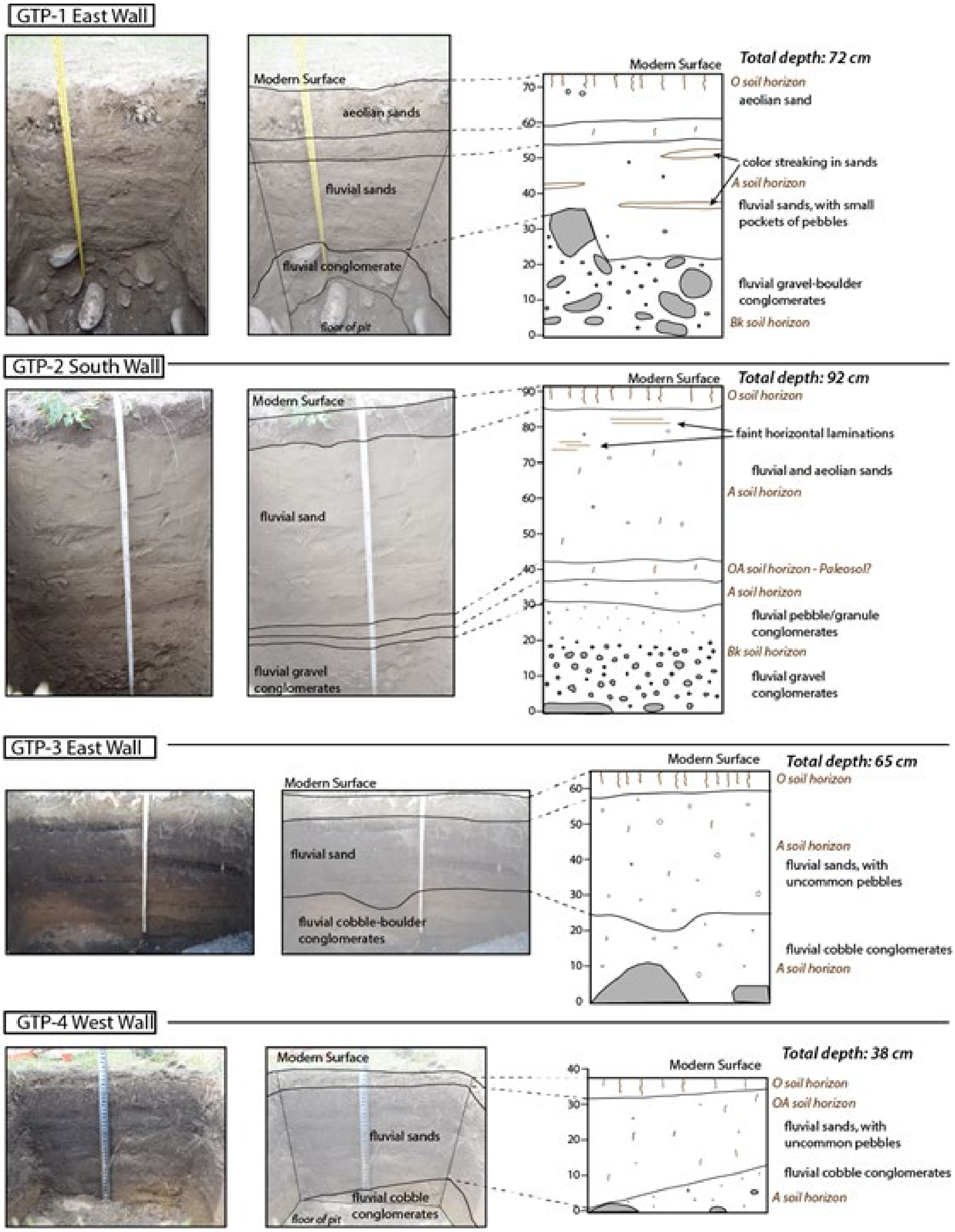

Fig. S2A.4. Field photos, interpretation, and schematic sketch of the Bagsagiin Bulan GTP1-4 test pits. Top – GTP-1, through bottom – GTP-4.

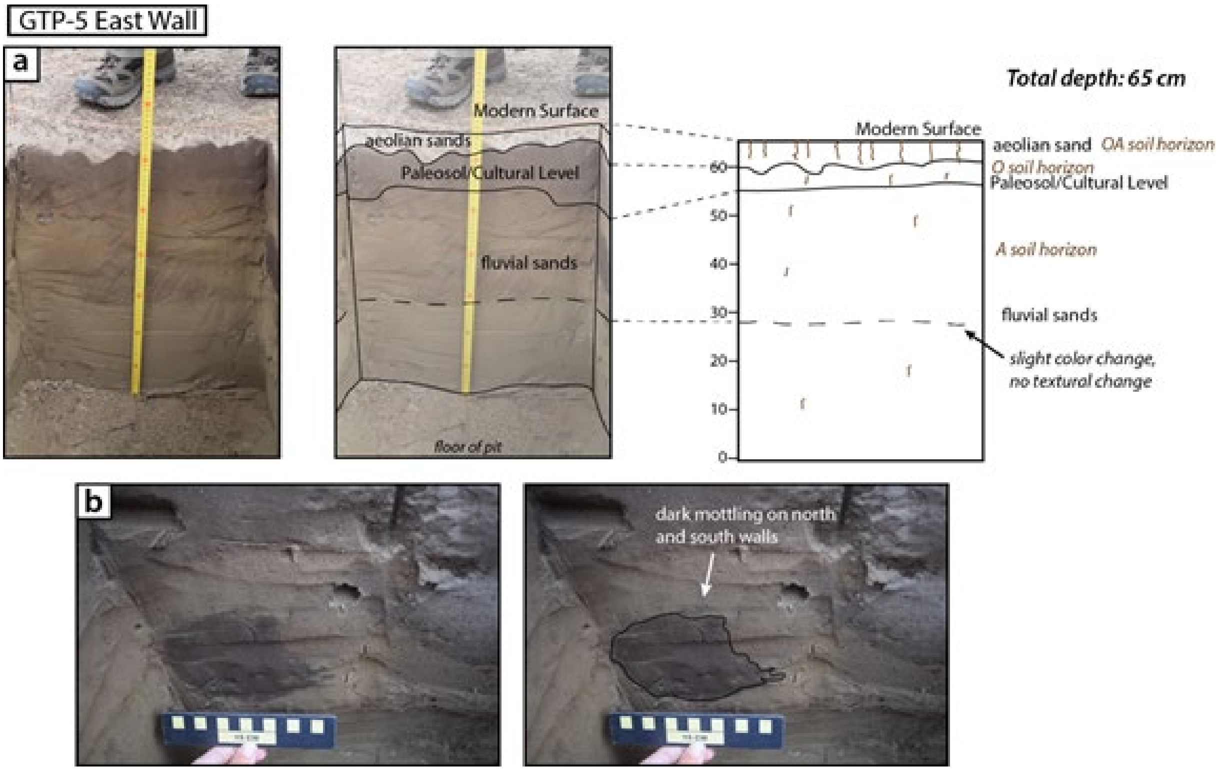

Fig. S2A.5. (a) Field photo, interpretation, and schematic sketch of the Bagsagiin Bulan GTP-5 test pit. (b) Field photo and interpretation of circular mottling observed in GTP-5.

Section S2B. Excavation plans and profiles

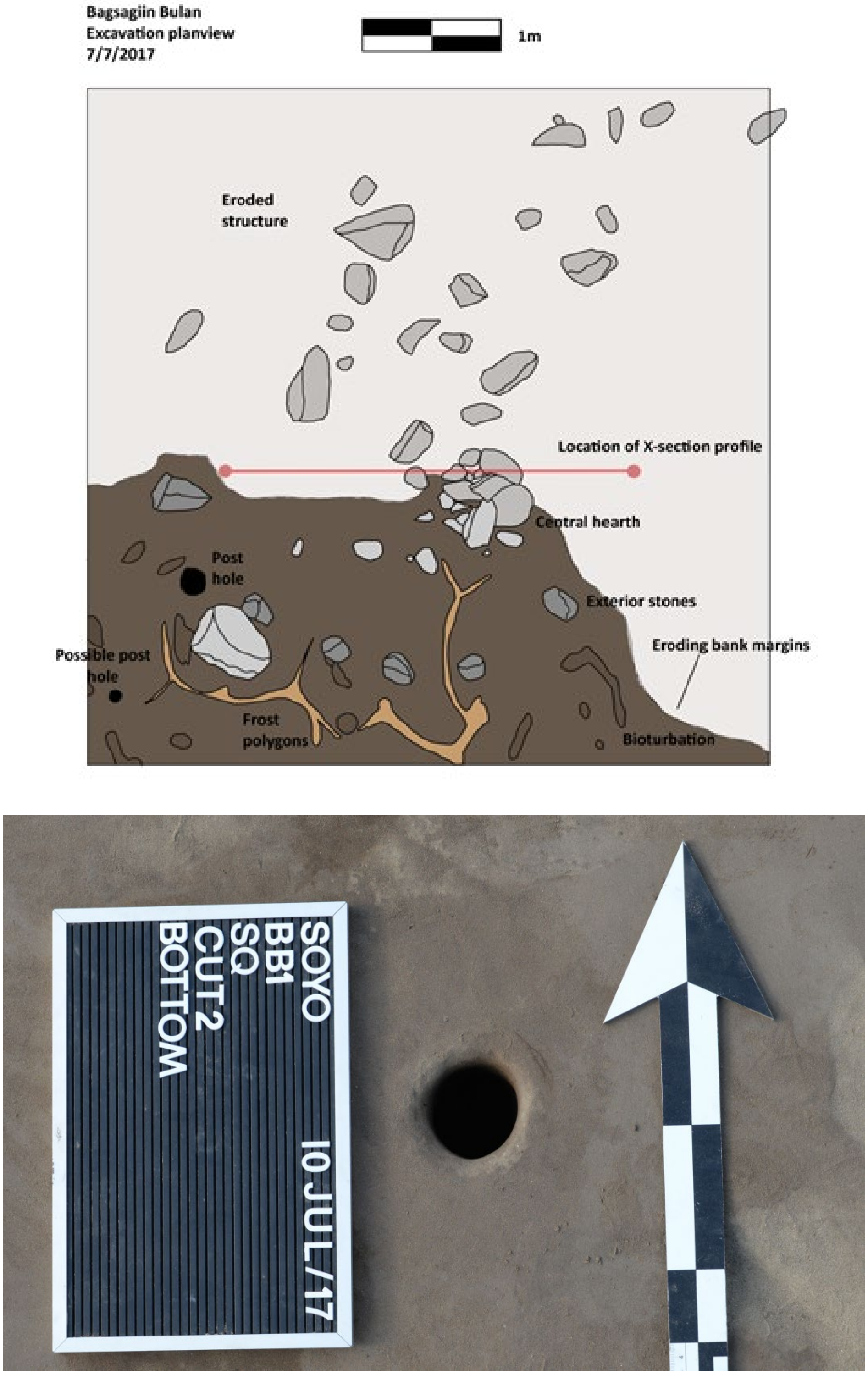

Figure S2B.1. A (left): Planview sketch of excavations at Bagsagiin Bulan, along with location of key features, bank margins, and cross-sectional profile. Soil colors are depicted using recorded Munsell color chart values. B (right): closeup of posthole following excavation.

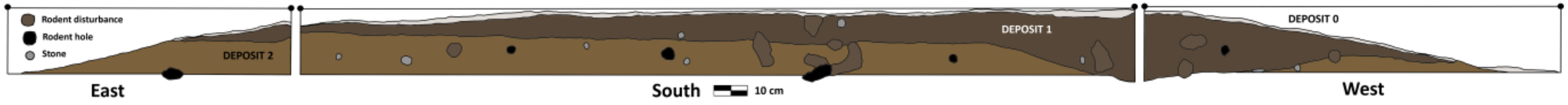

Figure S2B.2. Excavation profiles for Bagsagiin Bulan. Soil colors are depicted using recorded Munsell color chart values.

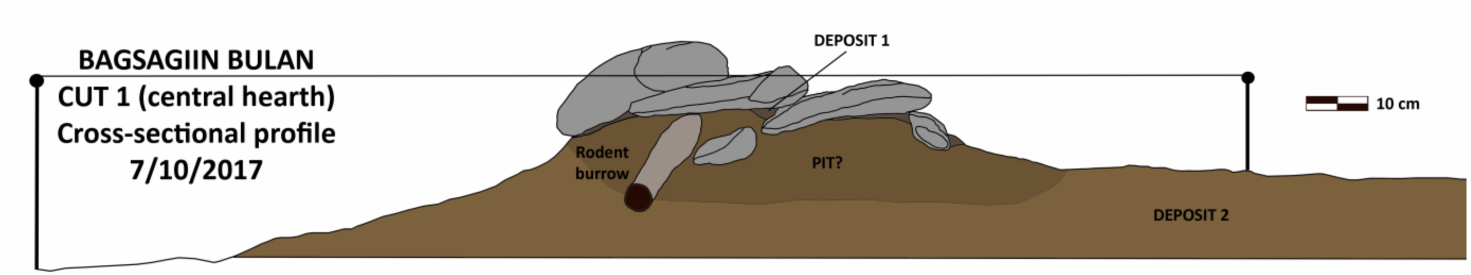

Figure S2B.3. Cross-sectional profile for Bagsagiin Bulan, showing soil color change suggestive of an ancient pit. Soil colors are depicted using recorded Munsell color chart values.

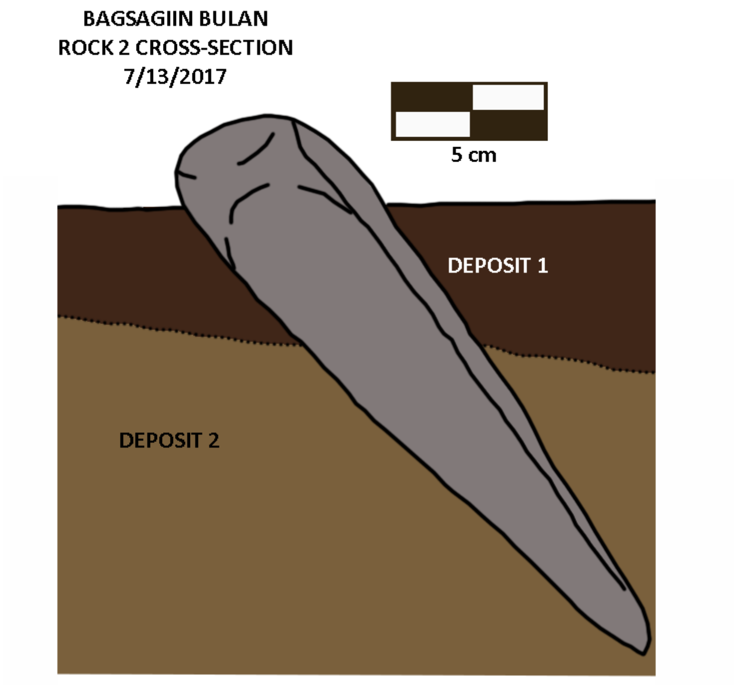

Figure S2B.4. Cross-sectional profile of structural stone 2 at Bagsagiin Bulan, showing that the stone was inset at a slightly acute angle directly into the underlying sand deposit, after which the paleosol (Deposit 1) formed, likely as a result of human activity and surface stability.Soil colors are depicted using recorded Munsell color chart values.

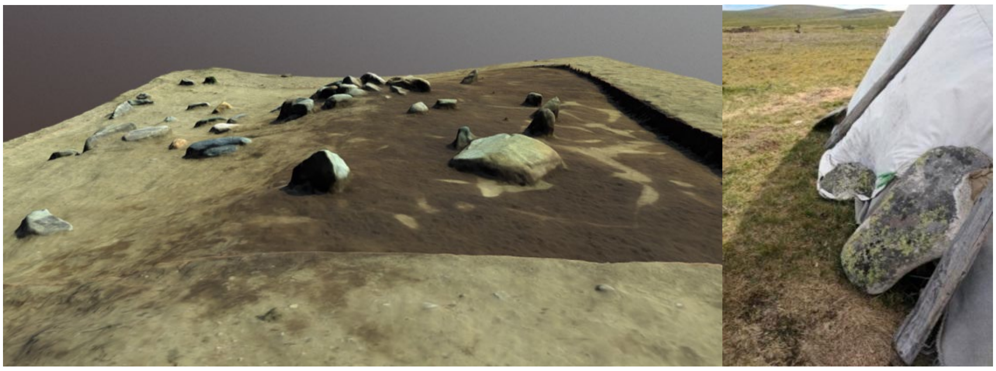

Figure S2B.5. 3D photogrammetry model showing large, flat stone laid against upright support stone in Bagsagiin Bulan (left), similar to the fashion that heavy stones are used to secure tent-pole *orts* habitations in northern Mongolia today among the Tsaatan/Dukha reindeer herders (right).

Section S2C. Artifacts

The lithic collection recovered from Bagsagiin Bulan totals 58 artifacts, including 21 pieces of debris (Table S2C) (Fig. S2C.1,6). According to preliminary, visual petrographic analysis, the majority of artifacts were made of flint. We identified one morphologically distinct core – a prismatic microblade core (Fig. S2C.1,7) . Core preparation blanks included one lateral spall, while in the category of detachments/spalls we identified 15 flakes (Fig. S2C.1,2) and 20 microblades (Fig. S2C. 1, 3 - 5).

Judging by the morphology of the available core and the composition of the artifact collection, the primary goal of the reduction sequence in this assemblage was microblade production. The blanks we recovered were extremely fragmented, with medial fragments predominating. Microblades show mostly unidirectional parallel dorsal scar patterns, with parallel lateral edges, trapezoidal to triangular midpoint cross sections, and straight longitudinal profiles. Most microblades have a reduced, linear or pointed striking platform. All microblades with a proximal edge show lips and lack percussion damage, and the angle of striking platform is around 90 degrees. It is important to note that the width of the blanks in the proximal and medial parts is similar. These characteristics are indicative of a pressure-flaking, unidirectional production system.

The tool kit consists of 2 tool types – end-scrapers, and microblades with ventral retouch. Our analysis shows that the industry of Bagsagiin Bulan was characterized by microblade pressure knapping. The issue of the emergence and using of these industries in northern Mongolia – Baikal region is a key subject of discussion. According to recent studies, the time of appearance of this technology can be identified around 15 ka (Rybin et. al. 2016), and they became widespread in the region during the Mesolithic/Neolithic. The results of this study allow us to suggest that pressure microblade technique remain in use across the early Bronze Age (ca. 2500 BCE), at least in northern Mongolia. Most likely, the late persistence of stone tool production reflects an inaccessibility / or local underdevelopment of metal production.

References cited

Rybin EP, Khatsenovich AM, Gunchinsuren B, Olsen JW, Zwyns N (2016) The impact of the LGM on the development of the Upper Paleolithic in Mongolia. Quat Int 425:69–87.

Table S2C. Lithic artifacts from Bagsagiin Bulan.

Artifact category	Counts/frequencies	
	N	%
Cores	1	1.7%
Pebbles	-	-
Core trimming elements	1	1.7%
Flakes (>20mm)	15	25.9%
Microblades	20	34.5%
Total non-debris artifacts	37	63.8%
Debris (chunks, chips, flakes less than 20mm)**	21	36.2%
**Total**	**58**	**100%**

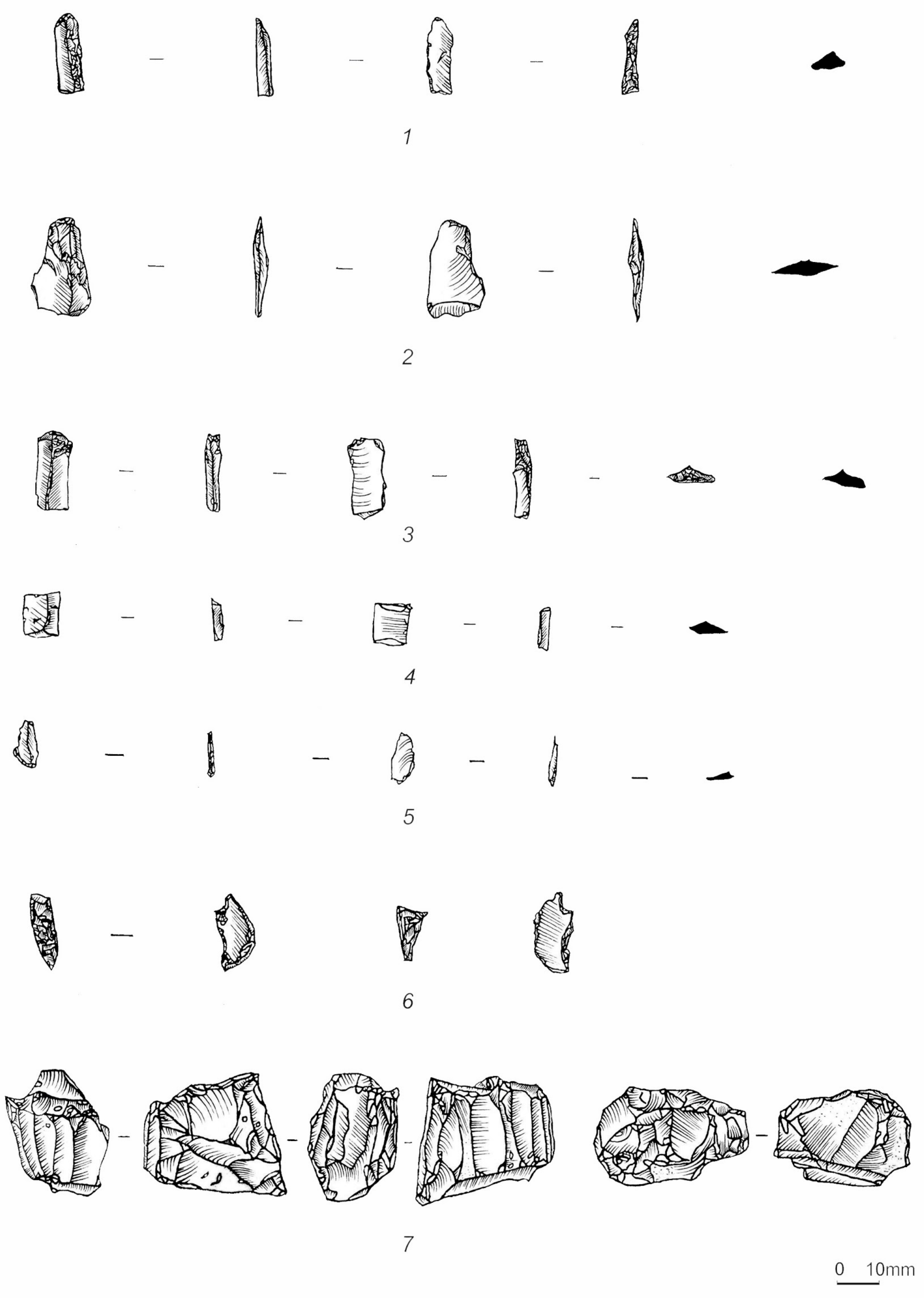

Figure S2C.1. Selected lithic assemblage from Bagsagiin Bulan. Drawings by V. Pham, and coloring by Olga Pugach. Recovered artifacts include microblades (1, 3-5), flakes (2), debris/shatter (6) and prismatic microblade core (7)

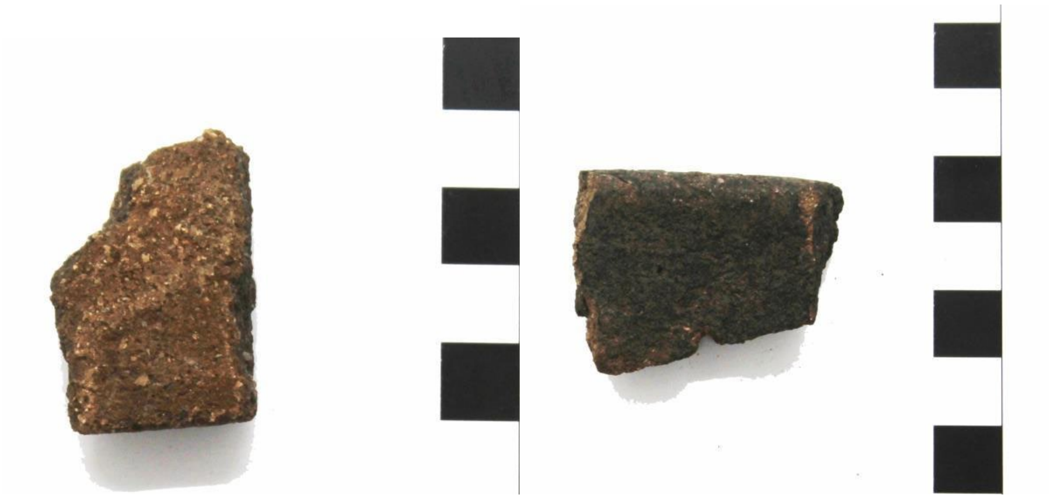

Figure S2C.2. Selected ceramic finds from the cultural level at Bagsagiin Bulan. Black bars on scale are spaced at 1 cm intervals.

Section S3. Biluut

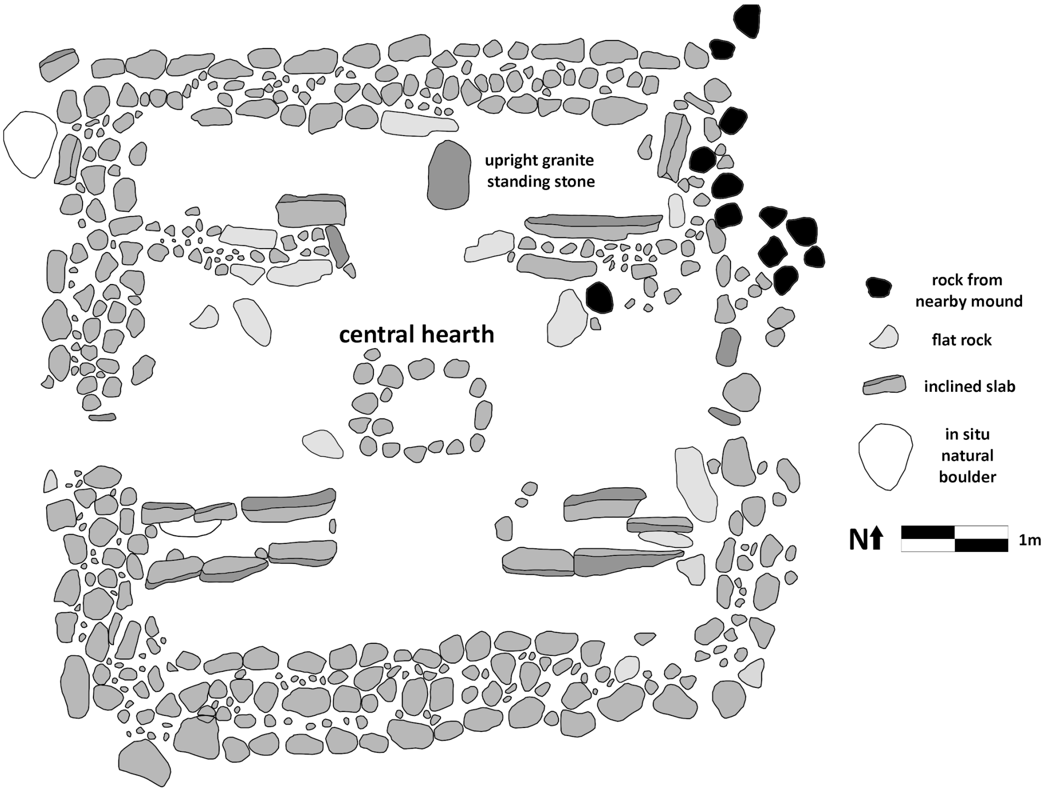

Figure S3.1. Planview excavation sketch of Biluut Peat Valley 3-3, modified from Fitzhugh and Kortum (2011).

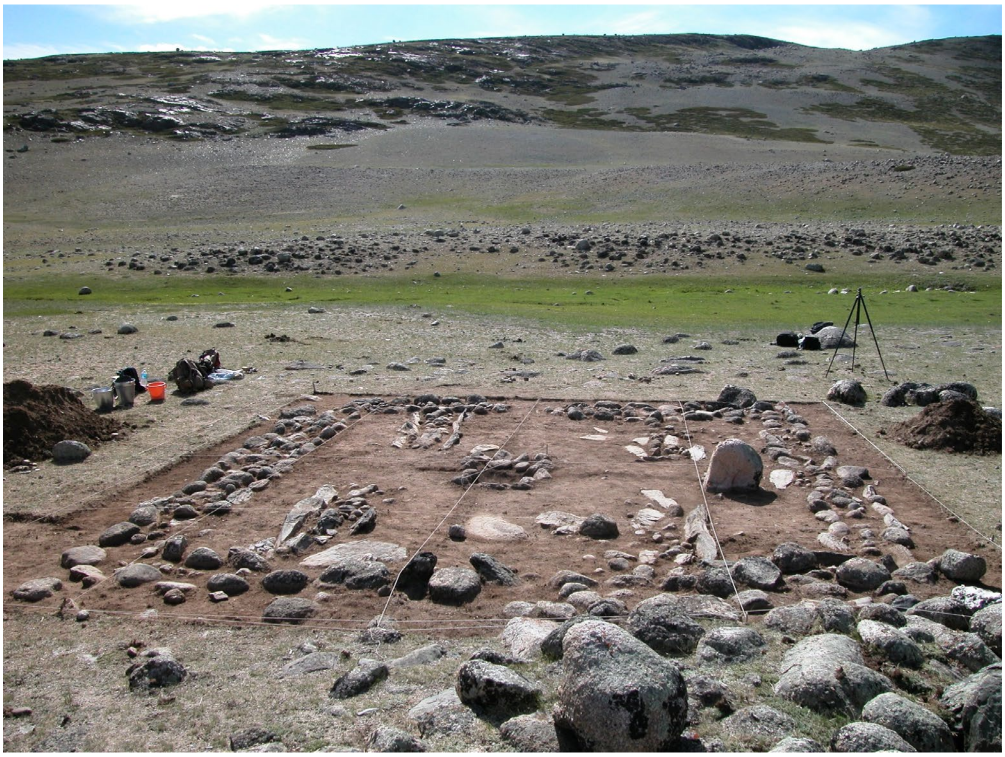

Figure S3.2 Peat Valley 3-3 at Biluut, during excavation in 2011. View to west. Photo: William Fitzhugh

Section S4. Tsagaan Asga.

Section S4A. Geomorphology of the Tsagaan Asga site.

Jessica Thompson Jobe, Department of Geology and Geological Engineering, Colorado School of Mines

The Tsagaan Asga site is located on a late Pleistocene terrace or glacial outwash plain and remnant MIS 4 or MIS 6 (>70 ka) lateral moraines (Lehmkuhl et al. 2016) along a large braided river, the Ikh Artstat Gol, which flows southward out of the Tsengel Khairkhan Mountains (Figure S4A.1). To the north and northeast of the site, large valleys formed by Pleistocene glaciers extend downward from the Tsengel Khairkhan Mountains (Lehmkuhl et al. 2016; Walther et al. 2017). Approximately 3 km upstream of the site, a complex of terminal and lateral glacial moraines from MIS 2 (>15 ka) occupy the river valley, and can be correlated to the preserved late Pleistocene glaciofluvial terrace downstream (Lehmkuhl et al. 2016; Walther et al. 2017), near the Tsagaan Asga site. Large parts of the glacial deposits are masked by a veneer of latest Pleistocene and Holocene aeolian sediments, deposited during late-glacial warming (Lehmkuhl et al. 2016). The archaeological site is located near a series of small ridges, called solifluction ridges, which formed during seasonal melting of the permafrost when water-saturated soil flows downhill. The ridges trend north-south and northeast-southwest and are superimposed on top of the glacial moraine remnants (Figure S4A.2). Approximately 1 km west of the site, a region of channel widening and aggradation was created directly upstream of a narrowing of the Ikh Artstat Gol as it flows between two bedrock outcrops. This channel widening and aggradation created a small lake within a field of thermokarst features on the highest late Pleistocene glaciofluvial terrace (Lehmkuhl et al. 2016).

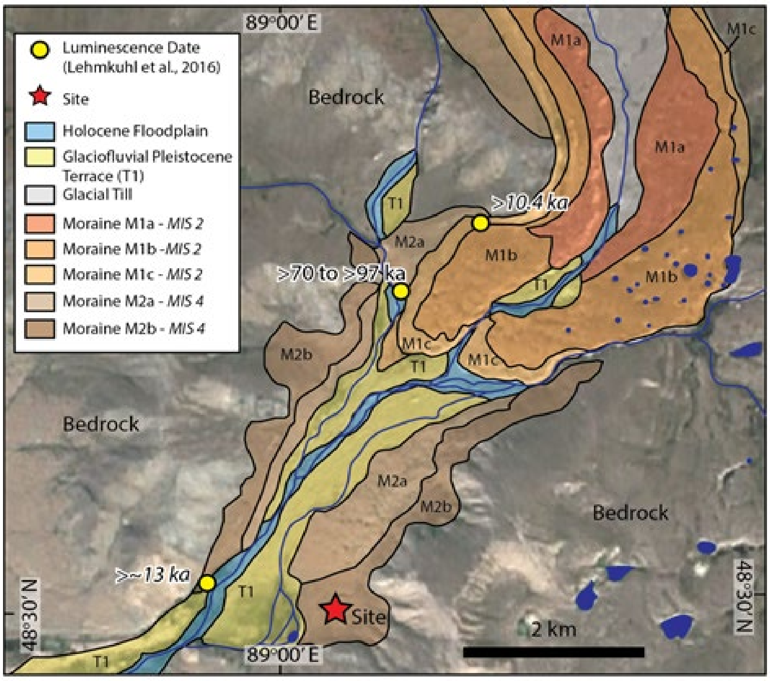

Figure S4A.1.Broader geomorphological setting for Tsagaan Asga, modified from Lehmkuhl et al., 2016.

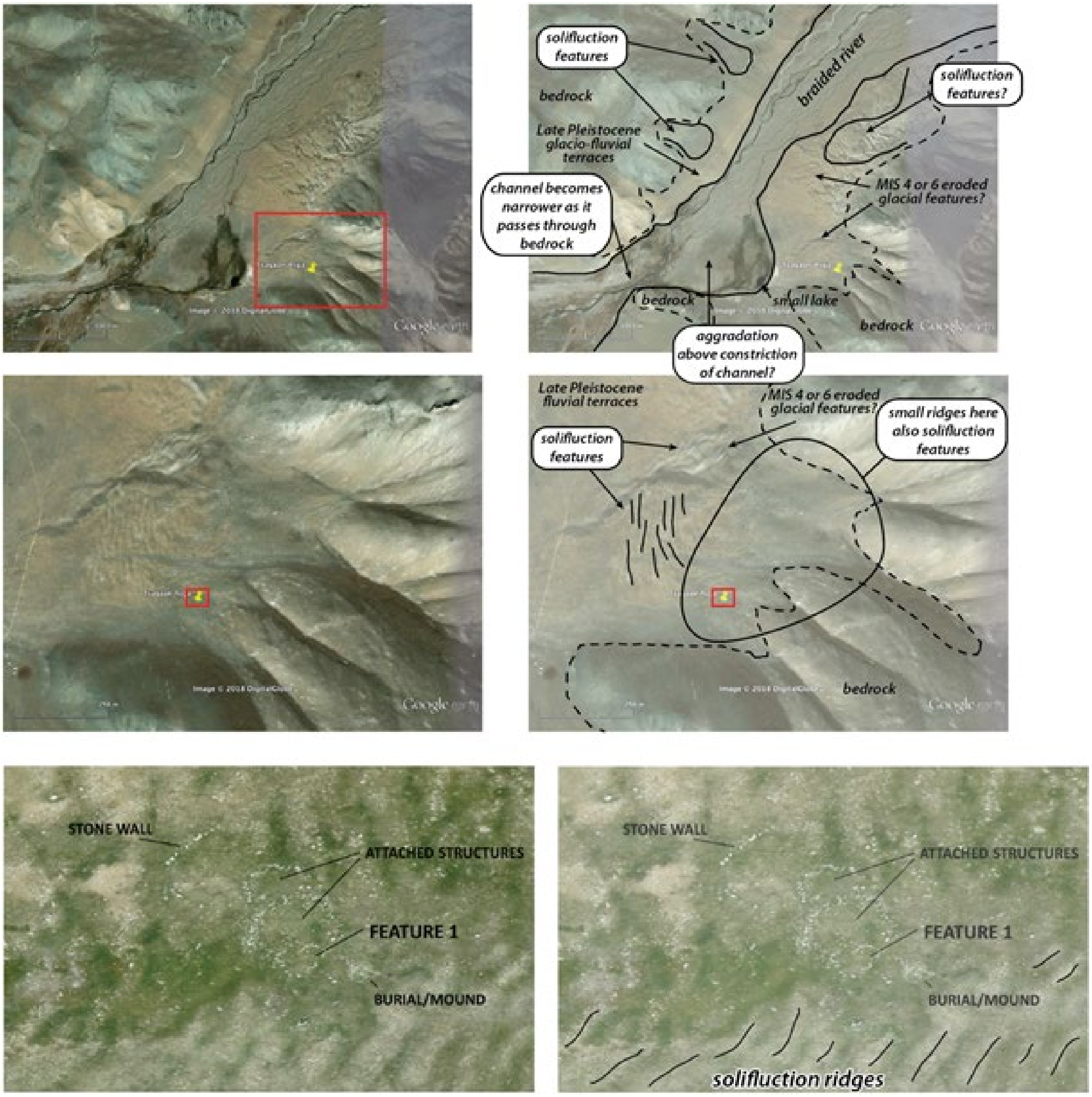

Figure S4A.2 GoogleEarth images and drone aerial photos illustrating solifluction ridges and local geomorphological features surrounding Structure 1 at Tsagaan Asga.

References:

Lehmkuhl F, Klinge M, Rother H, Hülle D (2016) Distribution and timing of Holocene and late Pleistocene glacier fluctuations in western Mongolia. Ann Glaciol 57(71):169–178.

Walther M, et al. (2017) Glaciers, Permafrost and Lake Levels at the Tsengel Khairkhan Massif, Mongolian Altai, During the Late Pleistocene and Holocene. Geosci J 7(3):73.

Section S4B. Excavation plans and profiles.

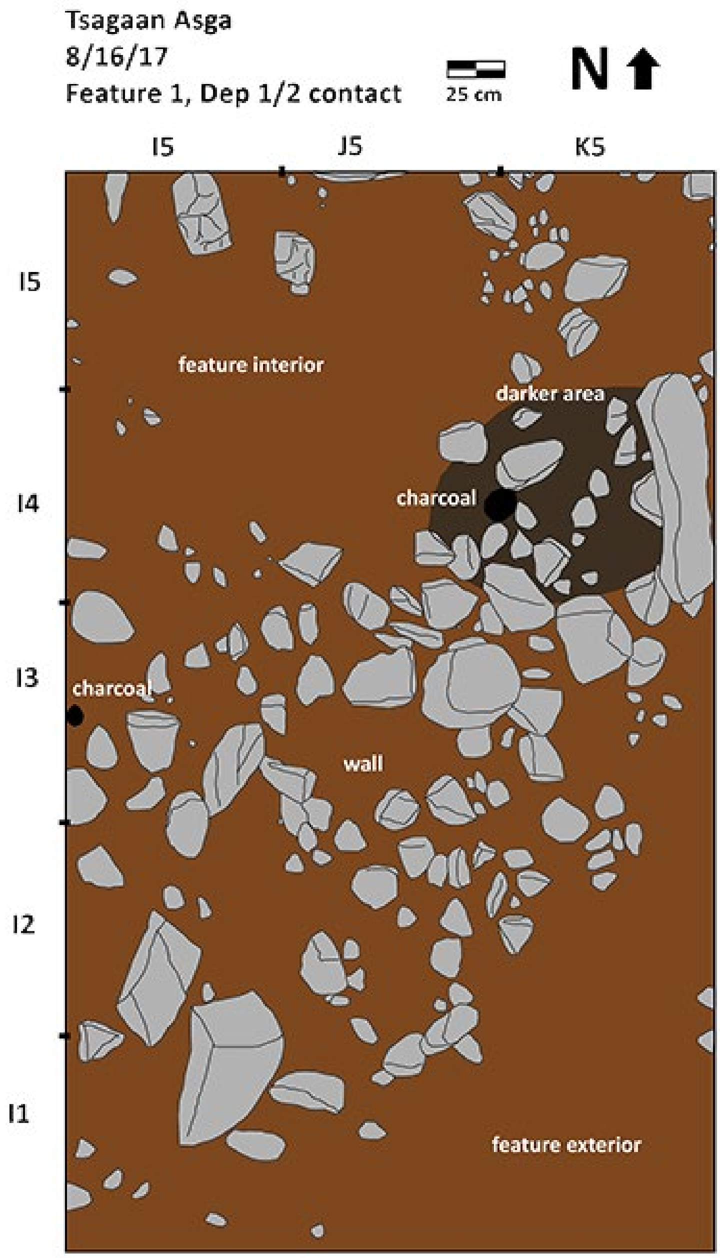

Figure S4B.1 Planview sketch of excavations at Tsagaan Asga, along with location of key features. Soil colors are depicted using recorded Munsell color chart values.

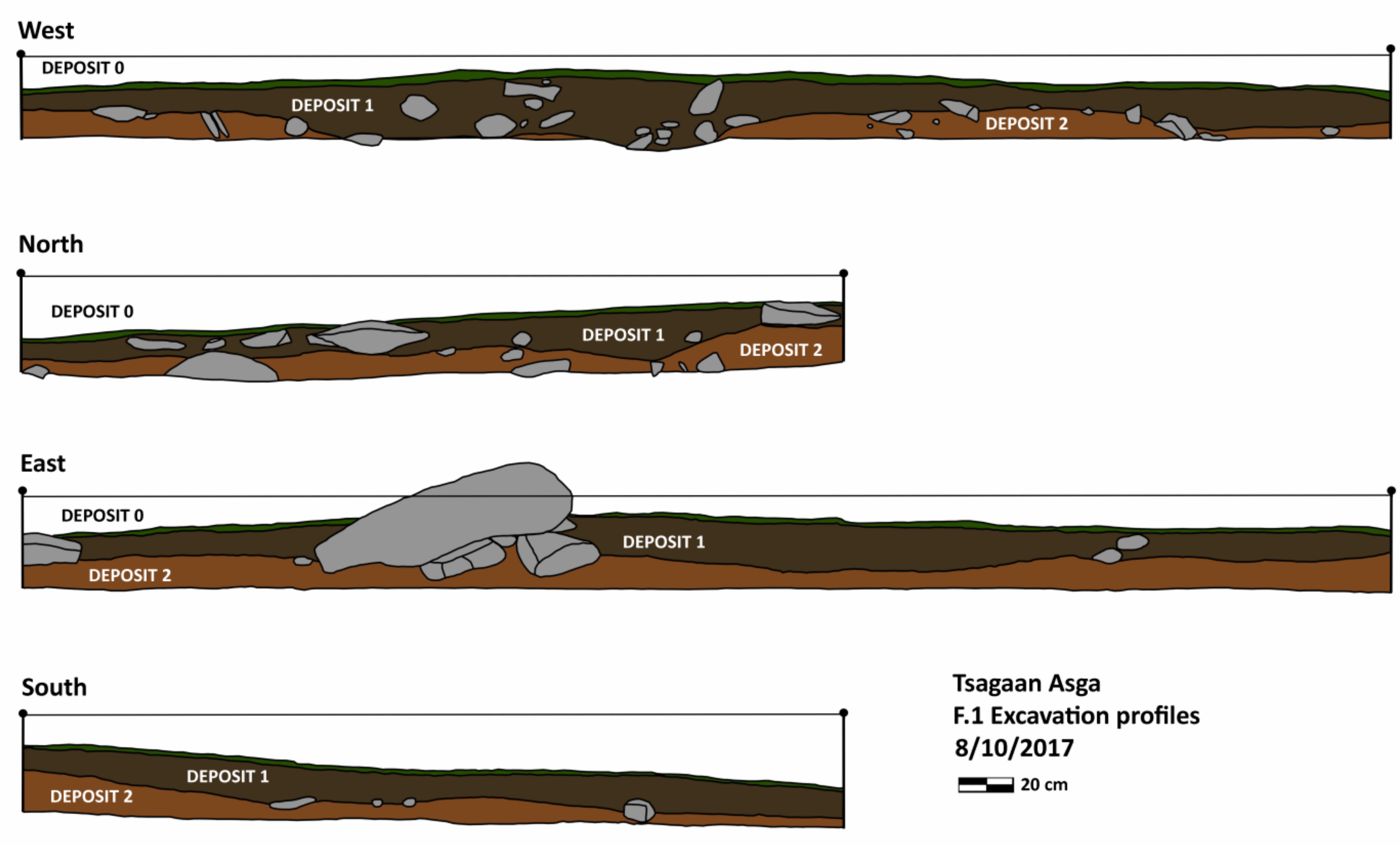

Figure S4B.2 Excavation profiles for Tsagaan Asga. West profile (top) shows that a shallow depression was excavated from previous soil surface (Deposit 2) to accommodate stone wall foundation.Soil colors are depicted using recorded Munsell color chart values.

Section S4C. Artifacts

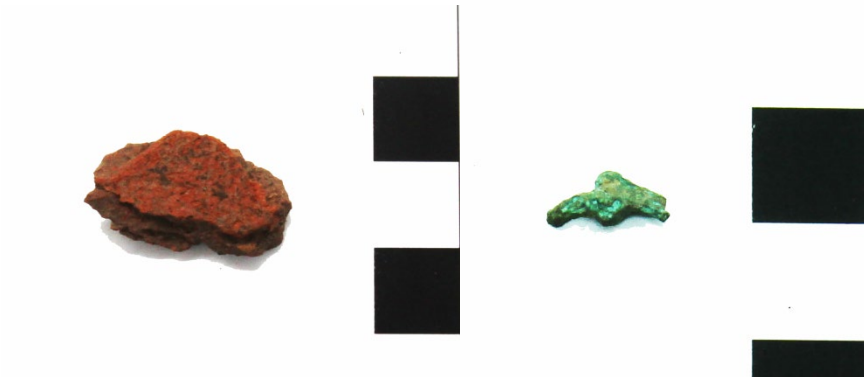

Figure S4C.1 Selected finds from the cultural level at Tsagaan Asga, including incised red ceramics (left), and bronze or copper slag (right).Black bars on scale are spaced at 1 cm intervals.

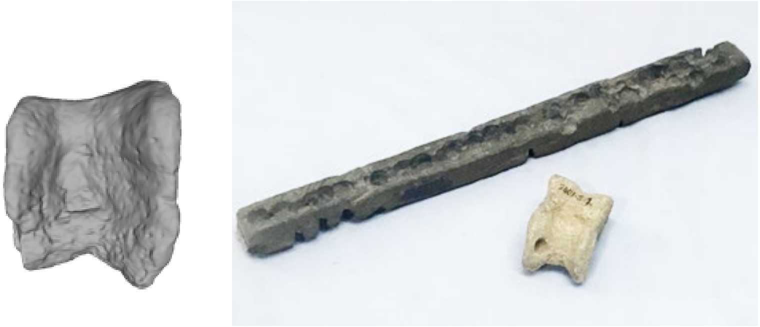

Figure S4C.2 Left: 3D scan of a heavily worn large mammal antler piece, identified as *Cervidae/Bovidae* using Zooarchaeology by Mass Spectrometry (ZooMS), left. The object has a circular depression in the center, as well as several thick cut marks in its exterior edges. Right: a firestarter kit in the ethnographic collections at the National Museum of Mongolia, consisting of a cow astragulus used to hold a bowdrill, and a grooved object serving as a base.

Section S5. Radiocarbon dates

**Sample name**	**Dated material**	**Context**	**Lab number**	**F^{14}C**	**± 1σ**	**% C**	**% N**	**δ^{13}C**	**δ^{15}N**	**C;N**	**^{14}C age**	**+/-**	**Cal. age range (2 σ)**
SOY-100146	Charcoal (wood)	Upper level, Deposit 1 (13-18 cmbd)	OxA-37217					-23.07			1974	25	40 cal. BCE-75 cal. CE
SOY-100261	Charcoal (wood)	Cut 1 (central hearth feature)	GrM-12150	0.6035	0.0009	55.6	--	-23.45 ±0.12	--	--	4056	15	2626-2495 cal. BCE
SOY-100113	Bone collagen (deer)	Surficial deposit (0-13 cmbd)	GrM-12148	0.9836	0.0012	42.7	15.2	-18.99 ±0.12	3.04 ±0.1	3.3	133	15	1684-1940 cal. CE
SOY-100212	Animal bone (*Capreolus sp.*)	Square Q9, surface (eroding)	OxA-37664					-19.38			40	26	modern
SOY-100232	Animal bone (cervidae/*Saiga* sp./ovinae)	Square O9 Deposit 1	OxA-37482					-19.73			135	26	1707-1919 cal. CE
TSA-100056	Bone collagen (*Ovis sp.*)	Square J4, (inside structure, atop Deposit 2)	GrM-12149	0.6624	0.0010	42.2	15.2	-17.90 ±0.12	9.49 ± 0.10	3.2	3308	15	1626-1530 cal. BCE
TSA-100052	Charcoal (wood)	Square I3, Deposit 1 (inside wall feature)	GrM-12151	0.6644	0.0010	59.6	--	9.49 ± 0.10	--	--	3285	15	1615-1516 cal. BCE

Section S6. Paleoethnobotanical Report for Bagsagiin Bulan and Tsagaan Asga

Robert N. Spengler III, Department of Archaeology, Max Planck Institute for the Science of Human History

This report presents archaeobotanical remains from two Bronze Age occupation sites in the Altai foothills of western Mongolia (Tsagaan Asga) and the Darkhad Basin of northern Mongolia (Bagsagiin Bulan). Taylor supervised the collection and flotation of sediment samples during excavations at the Bagsagiin Bulan and Tsagaan Asga sites. The samples were processed in the field using a basic bucket flotation method and sent to the Paleoethnobotany Laboratory at the Max Planck Institute for the Science of Human History in Jena, Germany, for analysis. The study comprises the first systematic archaeobotanical analysis in this part of Inner Asia. Due to extreme wind deflation, few attempts to collect ancient botanical remains have been successful in Mongolia, and we know little about the role of plants in the paleoeconomy of this massive geographic region. While the present assemblage only contains carbonized wood fragments and a few specimens of carbonized seeds from wild herbaceous plants, mostly *Chenopodium* spp., it is still an important contribution to Mongolian archaeology. The presence of ancient carbonized seeds demonstrates the potential for archaeobotanical recovery in the foothill zone of the eastern Altai Mountains.

In general, it is accepted that most Mongolian peoples through time had an economy that was heavily reliant upon pastoral products. The recovery of domesticated animal bones at the sites in this report attests to a pastoral component in the diet. Archaeological research across Mongolia has demonstrated that animals were present in the economy at least as far back in time as the third millennium BCE (Janz et al 2017). However, the deflated nature of most Mongolian archaeological sites precludes the possibility of interpreting the details of the paleoeconomy. Archaeobotanical studies prior to the Xiongnu period have failed to recover any plant remains, but the few studies that have been conducted at Xiongnu occupation sites have identified domesticated grain crops (e.g. Wright et al. 2007). Furthermore, opportunistic finds of grains from archaeological sites in the southern Altai suggest that cultivated crops may have been part of the economy by the first millennium BCE (Spengler et al. 2016). The recovery of grains in elite Xiongnu burials and iron and bronze farming tools from certain large-scale Xiongnu-period archaeological sites also opens the question of what role cultivated plants played in the economy by the end of the first millennium B.C. (Di Cosmo 1994). Beyond the question of cultivation, we currently have no data to discuss the role of foraging in the economy, despite the likely importance of wild plants in the past. Collectively, these data call for a more focused and systematic approach to the archaeobotany of Mongolia. The Bagsagiin Bulan and Tsagaan Asga archaeobotanical studies demonstrate that there is potential for future studies to tell us about the nature of plant use in Mongolia through time.

Methodology

Sediment samples were sorted in the field using a basic bucket-flotation method and the both heavy and light fraction portion of the samples were collected. A total of 15 samples was collected from Bagsagiin Bulan, and each sample consisted of 10 liters of sediment (totaling

150L). Six flotation samples were collected from Tsagaan Asga, each of which also consisted of 10 liters (totaling 60L of sediment). Once in the lab, samples were passed through nested U.S. geological sieves to ease sorting. Material smaller than 0.50 mm was not sorted. Carbonized wood fragments larger than 2.00 mm were counted. Seeds and seed fragments were separated from all sieve units. Only charred seeds were systematically collected, as uncarbonized seeds were extremely abundant and assumed to represent bioturbation, likely rodent activity. Paleoethnobotanist working around the world typically removed uncarbonized seeds, assuming they are modern and intrusive. The likelihood of rodent turbation is even greater in these steppe ecologies, despite the fact that several scholars assume the antiquity of uncarbonized seeds recovered from archaeological sites. Popova reported large numbers of uncarbonized *Chenopodium* and *Amaranthus* seeds at the Late Bronze Age site of Krasosomarskoe, in the Lower Volga Region in Samara, Russia (Anthony et al. 2005; Popova 2006). Shishlina et al. (2008:240-241) also reported uncarbonized *Amaranthus* seeds, specifically *Amaranthus albus*, at the site of Gashun-Sala in the Yergueni Hills, on the steppe, northwest of the Caspian Sea, in the Late Bronze and early Iron Age. Spengler (2013) noted large numbers of uncarbonized seeds (notably *Chenopodium*) in the upper excavation levels at the sites of Begash and Tuzusai in eastern Kazakhstan, but his attempt to date these seeds illustrated that they were modern intrusions. Other attempts to date uncarbonized seeds in Asian archaeobotany assemblages led to similar revelations (e.g. Leipe et al. 2017). It should also be noted that the majority of the uncarbonized seeds from these sites were from *Chenopodium* plants. The hard testae of *Chenopodium* seeds preserve well in most sediments; in addition, the seeds themselves are known to stay viable in the soil seed bank for decades.

Results

There are a total of 21 flotation samples in this study from two sites, with a total volume of floated sediment equaling 210 liters. A total of 152 carbonized seeds were recovered from that sediment, with a total density of 0.7 seeds per liter of sediment. Uncarbonized seeds were extremely abundant in these samples but were not collected under the assumption that they are modern intrusions, a supposition also supported by the copious amounts of rodent dung and modern plant rootlets. Both heavy and light fraction portions of the samples were analyzed. Of the 152 wild carbonized seeds, 151 of them come from the site of Bagsagiin Bulan, and only a single *Amaranthus* sp. seed and a carbonized fragment of an unidentifiable seed were recovered from Tsagaan Asga. Of the 151 wild carbonized seeds recovered from Bagsagiin Bulan, 150 of them were from *Chenopodium* plants. The samples from Bagsagiin Bulanalso contained thousands of uncarbonized seeds of *Chenopodium*. The other taxon of carbonized seeds at the site, with an n of 1, was *Polygonum* sp.

Wood

Wood fragments >2.00 mm were collected from all the samples. The fragments that were collected were too small for identification and no bark or identifiable parts were recovered. Eighty-three fragments of carbonized wood were recovered, of which 73 came from the Bagsagiin Bulan samples and 12 came from Tsagaan Asga.

Chenopodium Seeds in Eurasian Archaeobotany

The *Chenopodium* spp. seeds from Bagsagiin Bulan all have a characteristic embryo beak or radicle and rounded margins (Figure S6.1). *Chenopodium* seeds are some of the most commonly recovered seeds from both New and Old World macrobotanical assemblages. In some studies they are used to argue for disturbed anthropogenic environments, dung burning, agricultural weeds, or human foraging, depending what site they are recovered from and what arguments the researchers want to defend. However, it is important to note that these seeds are commonly recovered from assemblages across Central Asia and may simply represent random seed rain into the site. A single *Chenopodium* plant can produce over ten thousand seeds in one season; they are annuals and they grow rapidly. They are endozoochoric-dispersed and are often found in abundance near pastoralist sites. In fact, Spengler et al. (2013) pointed out that chenopod plants can be used as indicators of previous years′ herd animal pens and seasonal campsites. But chenopod seeds also have high

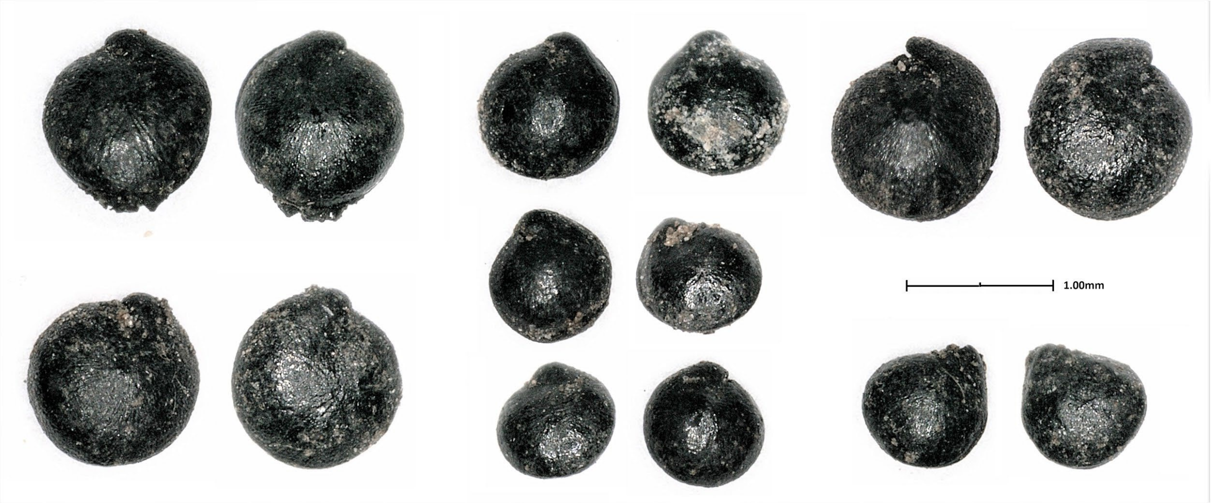

Figure S6.1 Ventral and Dorsal views of seven carbonized Chenopodium seeds from Bagsagiin Bulan. These images show the morphological variation present in the archaeobotanical specimens.

dormancy rates and can remain viable in the sediments for decades; therefore, they often make up the dominant seeds in the soil seed bank and are fast to recolonize an area after heavy grazing, fire, or flooding. Therefore, it is impossible to tell whether the carbonized seeds from Bagsagiin Bulanare the result of dung, sod, or brush burning, or even the burning of the sediments directly below the fire. They could represent modern dung or ancient sediment deposits that have simply preserved well; they could represent rodent cashes or sediment cracking and rootlet turbation. The possibility cannot be ruled out that they played a role in human dietary economy. It is also important to note that it is difficult to differentiate between carbonized and uncarbonized chenopod seeds, due to their dark seed coat, and some possible overlap may have occurred in the final counts. Therefore, I am cautious about interpretation of the significance of these seeds in the Bagsagiin Bulan assemblage.

Spengler (2013; Spengler et al. 2013; Spengler et al. 2017) found abundant remains of *Chenopodium* seeds at the sites of Tasbas, Begash, Mukri, and Tuzusai, all in the Semirech′ye region of eastern Kazakhstan. Based on the state of preservation and morphological characteristics I divided these remains into four categories: *Chenopodium album*-Type, *Chenopodium*-Other, *Chenopodium*-perisperm-only, and *Cheno-am* category. *Chenopodium* seeds are one of the most abundant and ubiquitous seeds in the Begash assemblage (n = 744); *Cheno-ams* are about as abundant (n = 663). Flad et al. (2009) did not differentiate wild seeds below family level, however, they do break Amaranthaceae and Chenopodiaceae into two separate groups at the site of Donghuishan in Gansu, dated between ca. 1550 and 1450 cal B.C. Eight grains of Chenopodiaceae were recovered from Bezumennoe 1 settlement in the Late Bronze Age of the Volga-Ural Region (Lebedeva 1996 discussed in Popova 2006). Shishlina et al. (2008) identified *Amaranthus album* at Gashun-Sala in the Caspian steppe. At the Late Shang period site of DGS PI HI, Fuller and Zhang (2007) found '*Chenopodium* cf. *album*'. Chenopodium seeds were also abundant in the high elevation medieval site of Tashbulak in Uzbekistan (Spengler et al. 2018).

At the site of Krasnosamarskoe in the Late Bronze Age, members of the Srubnaya Culture established small settlements with wooden structures (Anthony et al. 2005). Extensive archaeobotanical analysis at these sites produced no evidence of domestic crops. Popova (2006) argues for the importance of the wild grain *C. album* in the human diet (Popova 2006:307, 2007). High percentages of *C. album* were recovered from Peschanyi Dol 1, 2, and 3 (2 in particular), as well as at Krasnosamarskoe and Kibit 1 and 2 (Popova 2006: 265). A number of *Polygonum* nutlets were found in combination with *C. album* in a waterlogged pit (feature 10) at Krasnosamarskoe (Popova 2006:222-224). For discussions of the ethnographic use of *Chenopodium* seeds and greens in the Old World see Popova (2006:264); Boulos (1985:151); Luczaj and Szymañski (2007:14); Spengler (2017).

Spengler et al. (2013) conducted dung burning experiments in steppe ecologies and analyzed the resulting seed assemblage; they note that 50 percent of the assemblage was composed of chenopod seeds, despite the fact that they did not seed chenopod plants around where they collected the dung. Spengler (2017) recently synthesized a large amount of literature on dung burning evidence in the archaeological record. I noted that chenopod seeds are consistently high in these assemblages in response to their effective adaptation to endozoochoric dispersal. Iultimately suggested that most seeds do not survive the digestive process; therefore, because *Chenopodium* seeds preserve so well, they are concentrated by herd animals. This not only leads to higher densities of these plants growing in areas where herd animals graze or are penned, but it suggests that dung and dung burning assemblages will have much higher in chenopod seed counts. This is consistently the case in herding archaeological sites across Eurasia. Cautiously, this means that high levels of *Chenopodium* seeds in an archaeological assemblage can be used as an indicator or pastoralist activities and dung burning.

Conclusion

Archaeobotanists working at archaeological sites in the Eurasian steppes have often reported uncarbonized seeds in their assemblages. There are ongoing discussions about how and why this specific genus is so prominent in archaeobotanical assemblages in Eurasia, especially when it is not a steppe-adapted species. Scholars continue to debate over the possible role as a foraged grain or whether they are indicative of anthropogenic disturbance of the local paleoecology. However, Spengler (2017) has made a compelling argument that *Chenopodium*, *Amaranthus*, and *Polygonum* seeds are concentrated during the digestion process in herd animals and the ash from the burning of this dung is often very high in abundance of these taxa. There are not enough carbonized seeds in this study to make any sound conclusions about plant use at either of the sites. Due to the compicated nature of this specific genus in archaeobotanical assemblages, they cannot be used to determine paleoecological conditions in the region. However, this study does provide a preliminary paleoethnobotanical report allowing for future studies to build on. While no evidence for domesticated crops or indisputably foraged plants were recovered, it is important to keep in mind that the absence of evidence is not evidence for absence. Similar to other steppe archaeobotanical assemblages, carbonized *Chenopodium* seeds were the dominant category at Bagsagiin Bulan; while there are several possibilities to explain their introduction into the assemblage, the most likely scenarios are through the burning of dung as fuel or simply through background seed rain.

Mongolia BAGSAGIIN BULAN and TSAGAAN ASGA Archaeobotany									Amaranthaceae		Polygonaceae	Unidentifiable	Totals
Site	Trench	Context	Grid Coordinates	Comments	Sample #	Light and Heavy Fractions	Volume of Soil Floated (Liters)	Wood (Fragments > 2.oo mm) Ct.	*Chenopodium* spp.	*Amaranthus* sp.	*Polygonum* sp.	Unidentifiable Seed Fragments	Totals without Unident. Frags.
Mongolia	BAGSAGIIN BULAN	BB1	M8	Deposit 1	FS1	LF	10	13	74		1		75
		BB1	M8			HF		1					0
		BB1	M8	Deposit 2	FS2	LF	10		12				12
		BB1	M8			HF							0
		BB1	M9-1	Deposit 1	FS3	LF	10	2	41				41
		BB1	M9-1			HF							0
		BB1	N8	Deposit 0	FS4	LF	10	2	8				8
		BB1	N8			HF							0
		BB1	N9	Deposit 1	FS5	LF	10	5					0
		BB1	N9			HF							0
		BB1	O8	Deposit 0	FS6	LF	10	7					0
		BB1	O8			HF							0
		BB1	O8	Deposit 1	FS7	LF	10	7					0
		BB1	O8			HF							0
		BB1	O8	Deposit 2	FS8	LF	10	1	1				1
		BB1	O8			HF							0
		BB1	P8	Deposit 0	FS9	LF	10	5					0
		BB1	P8			HF							0
		BB1	P8	Deposit 1	FS10	LF	10	9	2				2
		BB1	P8			HF							0
		BB1	P9	Deposit 2	FS11	LF	10						0
		BB1	P9			HF							0
		BB1	O9/P9	Cut 1	FS12	LF	10	6	3				3
		BB1	O9/P9			HF							0
		BB1	Q8	Deposit 0	FS13	LF	10	3	4				4
		BB1	Q8			HF							0
		BB1	O9/10	Stone Structure	FS14	LF	10	8	5				5
		BB1	O9/10			HF							0
		BB1	O9/10	Cut 1	FS15	LF	10	2					0
		BB1	O9/10			HF		2					0
				**Subtotal**			150	73	150	0	1	0	151
	TSAGAAN ASGA	TSA	Feature 2	Deposit 1	FS16	LF	10	2		1		1	1
		TSA	Feature 2			HF							0
		TSA	I-4	Deposit 0	FS17	LF	10						0
						HF							0
		TSA	J-4	Deposit 1	FS18	LF	10						0
		TSA	J-4			HF							0
		TSA	J-5	Deposit 0	FS19	LF	10						0
						HF							0
		TSA	K-4	Deposit 1	FS20	LF	10	9					0
		TSA	K-4			HF							0
		TSA	K-5	Deposit 0	FS21	LF	10	1					0
		TSA	K-5			HF							0
				**Subtotal**			60	12	0	1	0	1	1
				**Totals**			210	85	150	1	1	1	152

Table S6. Archaeobotanical data for Tsagaan Asga (TSA) and Bagsagiin Bulan (BB1)

References Cited

Boulos L, Egypt C (1985) The Middle East. Plant Resources of Arid and Semiarid Lands: A Global Perspective:129–186.

Di Cosmo N (1994) Ancient Inner Asian Nomads: Their Economic Basis and Its Significance in Chinese History. J Asian Stud 53(4):1092–1126.

Flad R, Shuicheng L, Xiaohong W, Zhijun Z (2010) Early wheat in China: Results from new studies at Donghuishan in the Hexi Corridor. Holocene 20(6):955–965.

Fuller D, Zhang H (2007). A Preliminary Report on the Survey Archaeobotany of the Upper Ying Valley (Hennan Province). In *Dengfeng Wangchengang* (Great Elephant, Zhengzhou), pp. 916-958 [in Chinese].

Janz L, Odsuren D, Bukhchuluun D (2017) Transitions in Palaeoecology and Technology: Hunter-Gatherers and Early Herders in the Gobi Desert. Journal of World Prehistory 30(1):1–80.

Leipe C, et al. (2017) Barley (Hordeum vulgare) in the Okhotsk culture (5th-10th century AD) of northern Japan and the role of cultivated plants in hunter-gatherer economies. PLoS One 12(3):e0174397.

Łuczaj Ł, Szymański WM (2007) Wild vascular plants gathered for consumption in the Polish countryside: a review. J Ethnobiol Ethnomed 3:17.

Popova L (2006) Pastoralism during the Late Bronze Age in Russia: past interpretations and new goals for future research. Beyond the Steppe and the Sown: Proceedings of the 2002 University of Chicago Conference on Eurasian Archaeology, pp 459–468.

Shishlina NI (2008) Reconstruction of the Bronze Age of the Caspian steppes: life styles and life ways of pastoral nomads (British Archaeological Reports Ltd).

Spengler RN, et al. (2018) Arboreal crops on the medieval Silk Road: Archaeobotanical studies at Tashbulak. PLoS One 13(8):e0201409.

Spengler RN (2018) Dung burning in the archaeobotanical record of West Asia: where are we now? Veg Hist Archaeobot. doi:10.1007/s00334-018-0669-8.

Spengler RN, Ryabogina N, Tarasov PE, Wagner M (2016) The spread of agriculture into northern Central Asia: Timing, pathways, and environmental feedbacks. Holocene 26(10):1527–1540.

Spengler RN (2013) Botanical resource use in the Bronze and Iron Age of the central Eurasian mountain/steppe interface: Decision making in multiresource pastoral economies. Available at: http://openscholarship.wustl.edu/cgi/viewcontent.cgi?article=2045&context=etd.

Spengler RN, Frachetti MD, Fritz GJ (2013) Ecotopes and Herd Foraging Practices In the Steppe/Mountain Ecotone of Central Asia During the Bronze and Iron Ages. J Ethnobiol 33(1):125–147.

Wright J, Honeychurch W, Amartuvshin C (2009) The Xiongnu settlements of Egiin Gol, Mongolia. Antiquity 83(320):372–387

Section S7. DNA Metabarcoding, Biluut

Frederik Seersholm, TrEnD Laboratory, Curtin University

To test the DNA preservation under various degrees of calcination at Biluut Peat Valley 1, (Biluut 3-3), we analyzed five samples of 25 or 50 unidentifiable bone fragments using Bulk Bone Metabarcoding (Murray et al 2013). Samples characterised as unburnt (aDNA sample 1; 25 bones), partially burnt (aDNA sample 2-3; 50 bones) and burnt (aDNA sample 4-5; 50 bones) were tested. We found that all five bulk bone samples amplified at similar Ct values as the extraction blank and the no template control (see Table 1), indicating low or no endogenous DNA content in all samples. Sequencing of two mitochondrial barcodes targeting the 12S and 16S genes yielded a total of 16,921 and 40,942 DNA reads, respectively. In all but one sample, the most commonly identified species was *Homo sapiens,* which is a well-known contaminant in ancient DNA studies, due to trace amounts of human DNA in many laboratory reagents (Leonard et al. 2007). Furthermore, in sample 2 and 3 we detected DNA from two other common contaminants, *Gallus gallus* and *Sus*. In sample 4, on the other hand, no human DNA was detected with the 12S assay, instead DNA from *Bos* was identified. The absence of human DNA together with the detection of one of the previously identified species at Peat Valley 1, could indicate that sample 4 still holds some endogenous DNA. However, *Bos* have also previously been identified as a laboratory contaminant, and the 16S assay did not yield any DNA from cattle in Sample 4. Hence, we conclude that no DNA is preserved from sample 1-3 and 5, while sample 4 could hold trace amounts of endogenous DNA from cattle.

Methods

For ancient DNA analysis one sample of 25 bones (aDNA sample 1) and four samples of 50 bones each (aDNA sample 2-5) from Biluut Peat Valley 1, were analysed with Bulk Bone Metabarcoding. For DNA extractions, each sample was ground using a Retsch PM200 Planetary Ball Mill at 400 rpm and ~150mg of the resulting bone powder was incubated overnight in digestion buffer (0.25 mg Proteinase K + 1 mL 0.5 m EDTA) at 55°C. Next, samples were spun down and the supernatant was concentrated to 50 µL in a MWCO 30,000 Vivaspin 500 column (Sigma-Aldrich). Lastly, the concentrate was purified in a MinElute PCR Purification column (Qiagen) using a modified binding buffer (40% Isopropanol, 0.05% µL Tween 20, 90 nm NaAc and 5M GuanHydCh in Ultrapure water) (Dabney et al 2013).

Metabarcoding was carried out using two mitochondrial assays targeting mammals (Mam16S(Taylor 1996)) and all vertebrates (12SV5(Riaz et al 2011)), respectively. For each sample/assay combination PCR reactions were setup with 1 µL of template DNA, 1X buffer (ThermoFisher), 2mM MgCl2 0.25mM dNTPs, 1U AmpliTaq Gold® DNA Polymerase, 0.6 µL 5X SYBR green, 0.4 mg/ml BSA and 0.4 µM of primers in 25µL reactions. PCR cycling conditions consisted of an initial denaturation for 10min at 95°C followed by 50 cycles of 30s at 95°C, 30s at 54°C and 45s at 72°C, and a final elongation step of 10min at 72°C. Next, PCR reactions were cleaned up using the QiaQuick PCR purification kit (Qiagen) following the manufacturer's instructions and pooled for sequencing. Followingly. PCR products were sequenced on the Illumina MiSeq platform using the 300-cycle MiSeq Reagent Standard Kit v2 (Illumina) with custom sequencing primers.

Bioinformatics processing of the raw sequencing reads was carried out using a custom made pipeline described in Seersholm et al.(2018). In short, reads were filtered, demultiplexed and denoised using OBItools(Boyer et al 2016) and sumaclust. Next, chimeras were removed using vsearch (vsearch --uchime_denovo)(Rognes et al 2016), and lastly, each filtered read was queried against the NCBI nt database (ftp://ftp.ncbi.nlm.nih.gov/blast/db/nt*gz) (Benson et al 2006) using megablast (Altschul et al 1990). Lastly, taxonomic assignments were carried out using the script blast_getLCA.py (https://github.com/frederikseersholm/blast_getLCA).

	**16S**			**12S**		
	Ct value	**n reads**	**Species ID**	**Ct value**	**n reads**	**Species ID**
Sample 1	37.29	6960	Homo sapiens	38.13	3400	Homo sapiens
Sample 2	39.75	10176	Homo sapiens	36.52	4318	Homo sapiens, Gallus gallus, Sus scrofa
Sample 3	42.96	3103	Homo sapiens	36.47	4746	Homo sapiens, Gallus gallus
Sample 4	38.39	10196	Homo sapiens	43	1616	Bos sp.
Sample 5	37.71	10507	Homo sapiens	38.4	2841	Homo sapiens
Extr. blank	38.94	7088	Homo sapiens	NA	NA	NA

NTC	46.56	0	NA	NA	NA	NA

Table S7.1. Sequencing information for Bulk Bone Metabarcoding from Biluut Peat Valley 1, Feature 3-3.

References cited

Murray DC, et al. (2013) Scrapheap challenge: a novel bulk-bone metabarcoding method to investigate ancient DNA in faunal assemblages. *Sci Rep* 3:3371.

Leonard JA, et al. (2007) Animal DNA in PCR reagents plagues ancient DNA research. *J Archaeol Sci* 34(9):1361–1366.

Dabney J, et al. (2013) Complete mitochondrial genome sequence of a Middle Pleistocene cave bear reconstructed from ultrashort DNA fragments. *Proc Natl Acad Sci U S A* 110(39):15758–63.

Taylor PG (1996) Reproducibility of Ancient DNA Sequences from Extinct Pleistocene Fauna. *Mol Biol Evol* 13(1):283–285.

Riaz T, et al. (2011) ecoPrimers: inference of new DNA barcode markers from whole genome sequence analysis. *Nucleic Acids Res* 39(21):e145.

Seersholm F V., et al. (2018) Subsistence practices, past biodiversity, and anthropogenic impacts revealed by New Zealand-wide ancient DNA survey. *Proc Natl Acad Sci*:201803573.

Boyer F, et al. (2016) obitools: A unix-inspired software package for DNA metabarcoding. *Mol Ecol Resour* 16(1):176–182.

Rognes T, Flouri T, Nichols B, Quince C, Mahé F (2016) VSEARCH: a versatile open source tool for metagenomics. *PeerJ* 4:e2584.

Benson DA, Karsch-Mizrachi I, Lipman DJ, Ostell J, Wheeler DL (2006) GenBank. *Nucleic Acids Res* 34:16–20.

Altschul SF, Gish W, Miller W, Myers EW, Lipman DJ (1990) Basic local alignment search tool. *J Mol Biol* 215(3):403–410.
